# Detection of Slipped-DNAs at the Trinucleotide Repeats of the Myotonic Dystrophy Type I Disease Locus in Patient Tissues

**DOI:** 10.1371/journal.pgen.1003866

**Published:** 2013-12-19

**Authors:** Michelle M. Axford, Yuh-Hwa Wang, Masayuki Nakamori, Maria Zannis-Hadjopoulos, Charles A. Thornton, Christopher E. Pearson

**Affiliations:** 1 Genetics & Genome Biology, The Hospital for Sick Children, Toronto, Ontario, Canada; 2 Department of Molecular Genetics, University of Toronto, Toronto, Ontario, Canada; 3 Department of Biochemistry, Wake Forest University School of Medicine, Winston-Salem, North Carolina, United States of America; 4 Department of Neurology, University of Rochester School of Medicine and Dentistry, Rochester, New York, United States of America; 5 Goodman Cancer Research Centre and Department of Biochemistry, McGill University, Montreal, Quebec, Canada; University of Florida, United States of America

## Abstract

Slipped-strand DNAs, formed by out-of-register mispairing of repeat units on complementary strands, were proposed over 55 years ago as transient intermediates in repeat length mutations, hypothesized to cause at least 40 neurodegenerative diseases. While slipped-DNAs have been characterized *in vitro*, evidence of slipped-DNAs at an endogenous locus in biologically relevant tissues, where instability varies widely, is lacking. Here, using an anti-DNA junction antibody and immunoprecipitation, we identify slipped-DNAs at the unstable trinucleotide repeats (CTG)n•(CAG)n of the myotonic dystrophy disease locus in patient brain, heart, muscle and other tissues, where the largest expansions arise in non-mitotic tissues such as cortex and heart, and are smallest in the cerebellum. Slipped-DNAs are shown to be present on the expanded allele and in chromatinized DNA. Slipped-DNAs are present as clusters of slip-outs along a DNA, with each slip-out having 1–100 extrahelical repeats. The allelic levels of slipped-DNA containing molecules were significantly greater in the heart over the cerebellum (relative to genomic equivalents of pre-IP input DNA) of a DM1 individual; an enrichment consistent with increased allelic levels of slipped-DNA structures in tissues having greater levels of CTG instability. Surprisingly, this supports the formation of slipped-DNAs as persistent mutation products of repeat instability, and not merely as transient mutagenic intermediates. These findings further our understanding of the processes of mutation and genetic variation.

## Author Summary

Over 30 diseases are caused by the expansion of a trinucleotide repeat (TNR) in a specific gene, including the most common adult-onset form of muscular dystrophy, myotonic dystrophy (DM1). The mechanistic contributors to this unstable (TNR) expansion are not fully known, although since the discovery of these types of diseases over twenty years ago, the extrusion of the expanded repeats into mutagenic slipped-DNA conformations has been hypothesized. Here, we show the presence of slipped-DNA at the DM1 disease locus in various patient tissues. The allelic amounts of slipped-DNA in tissues correlate with overall levels of repeat instability. Slipped-DNA was also found to form in clusters along a tract of expanded repeats, which has been previously shown *in vitro* to impede DNA repair. This is the first evidence for slipped-DNA formation at an endogenous disease-causing gene in patient tissues.

## Introduction

**Figure 1:**
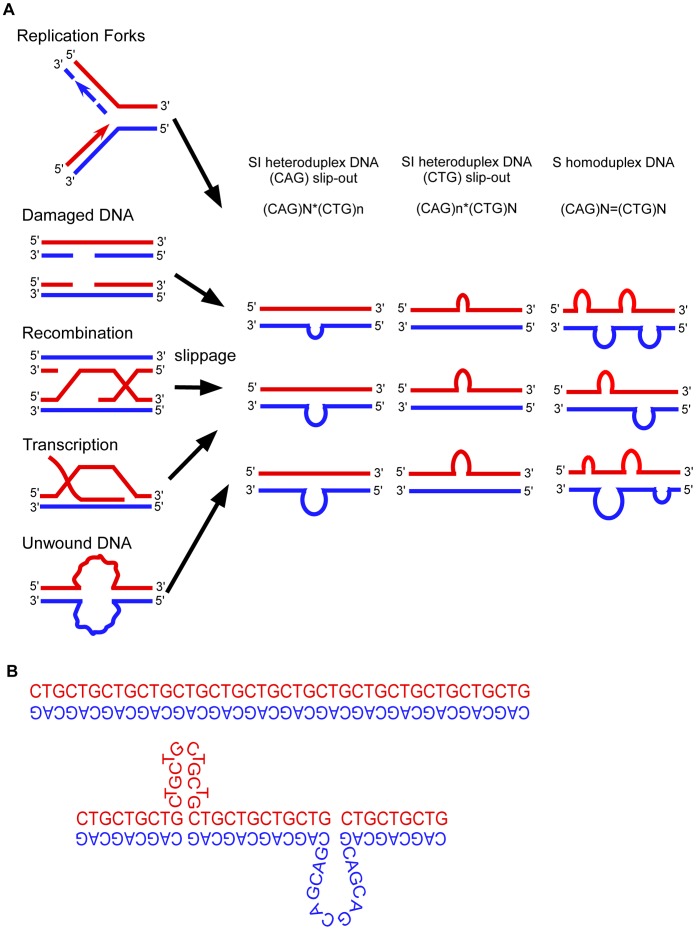
Models of expansion of trinucleotide repeats. (A) Slipped-strand DNAs can form during various metabolic processes such as replication, repair, recombination, transcription, and at unwound DNA. Slipped-out- DNAs may form on either the CTG or CAG strand, forming SI-DNA heteroduplexes or S-DNA homoduplexes. S-DNA contains the same number of repeats in both DNA strands, with multiple clustered slip-outs per molecule. SI-DNA contains differing numbers of repeats in each strand. Mispairing of the repeats are shown at right. (B) Model of out-of-register DNA slippage in trinucleotide repeats. Slippage and mis-pairing of triplet repeats by the complementary repeat units shifting out-of register, leading to slipped-out repeats.

All models proposed to explain the instability of trinucleotide repeats involve DNA slippage at the repeats (Fig. 1) [1]–[12]. Slipped-DNAs were first hypothesized to exist in 1958 [13]. Slipped-DNAs are thought to contribute to more than 30 neuromuscular/neurodegenerative diseases caused by unstable microsatellite repeats, including myotonic dystrophy type 1 (DM1) and numerous cancers that show microsatellite instability [1]–[3], hence understanding slipped-DNAs in patient tissues is of great importance [14], [15]. Expansion mutations continue in DM1 patients as they age, coinciding with worsening symptoms. Patients exhibit inter-tissue repeat length differences as great as 5,770 repeats, with large expansions occurring in affected tissues such as brain, muscle and heart, indicating high levels of continuing expansions [4], [5]. The formation and aberrant repair of slipped-DNAs is a likely source of repeat instability and progressive disease severity in patients (Fig. 1) [6], [7]. An understanding of these DNA mutagenic intermediates in patients should provide insight as to how they may be processed and lead to mutations. The important questions demanding answers are 1) Do slipped-DNAs form at disease loci? 2) Do their levels vary in patient tissues that undergo variable levels of repeat expansion within a given individual? And, 3) What is the biophysical structure of these slipped-DNAs? These questions cannot be answered in a heterologous model system that shows repeat instability that does not reflect the instability ongoing in a patient, nor one lacking tissues. While slipped-DNAs have been characterized *in vitro* [8], [9], data supporting the presence of slipped-DNAs at an endogenous locus in biologically relevant tissues has been lacking. Previously it was reported that hairpin DNAs can form in a model cell line which contain CTG/CAG repeats integrated at an ectopic locus, where instability was contraction biased as opposed to the expansion bias present in affected patients (10). Although the authors report hairpin formation in living cells, that report could not comment upon either structure formation at an endogenous disease locus, their variation between tissues showing variable instability, upon their persistence in patients, or on the structural features of the DNAs. Furthermore, in that system, instability and hairpin detection depended upon DNA replication, contrasting with the high levels of instability arising in post-mitotic tissues of patients. It is imperative to study instability in patient tissues since repeat mutations are expansion-biased, arising by processes distinct from contractions, coupled with the known locus-specific effects, the tissue-specific variations of instability, and the fact that most expansions arise in non-replicating cells. To identify slipped-DNAs at a disease locus in patient tissues we devised a DNA-immunoprecipitation (IP) protocol that uses a highly specific monoclonal anti-DNA junction antibody (2D3) that recognizes 3-way DNA junctions [9], [11], [12], a structural feature of slipped-DNAs [8], [9]. The 2D3 antibody has been characterized extensively (summarized in Text S1 and citations therein). Briefly, 2D3 binds specifically to junction-containing DNAs with no sequence preference. Footprinting has mapped the 2D3 binding site at DNA junctions [9], [11], [12] where binding can occur from either of the angles sub-tended by the junction arms. For each junction 1–2 antibodies can bind per DNA junction. 2D3 does not bind single-stranded DNA, hairpins, Z-DNA, triplex or quadruplex, with no off-target binding reported in *in vitro* systems used (see Text S1 and citations therein). 2D3 binds best to slipped-DNAs [9], strengthening its use to isolate these structures.

## Results

**Figure 2:**
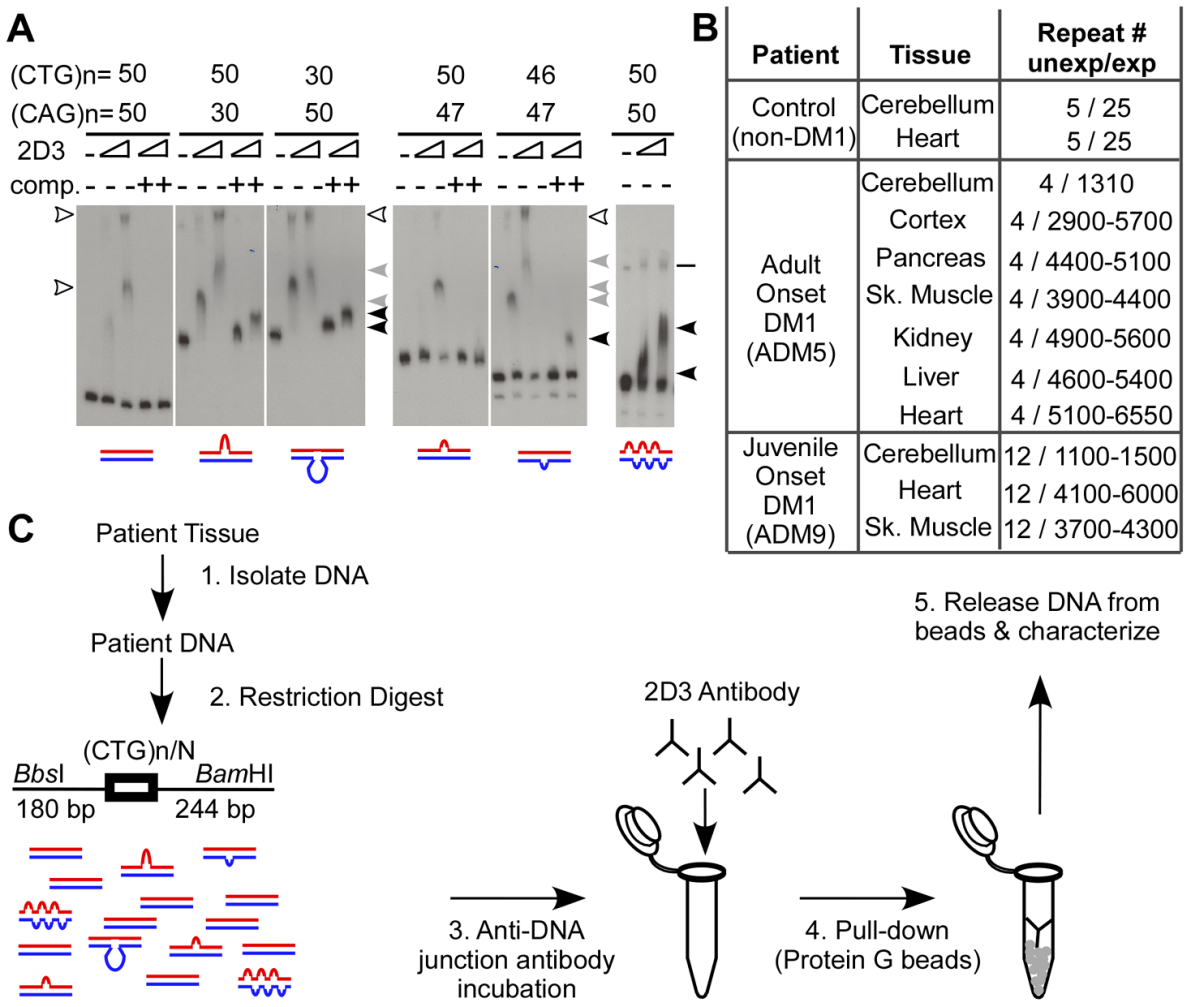
Slipped-DNAs are bound by anti-DNA junction antibody. (**A**) The anti-DNA junction antibody 2D3 bound slip-outs of 1-, 3- and 20-excess repeats as well as homoduplex slipped-DNAs with multiple clustered slip-outs/molecule by electrophoretic mobility shift assay. DNA substrates were 59 bp+(CT/AG)n+54 bp radiolabeled, gel-purified and used in binding experiments. Arrowheads indicate non-specific, specific and competition-resistant specific complexes. Line for lanes of S-DNA indicates a non-specific DNA. Triangles indicate increased antibody; + indicates addition of non-specific (plasmid) competitor DNA. All samples of the band-shift experiment were resolved on a single gel with panels separated for clarity. See Fig. S2 for control IgG_1_ Ab binding. (**B**) DM1 post-mortem patient and control, tissue, and DM1 CTG tract sizes (for the non-expanded and expanded allele for the patients, and both non-expanded alleles for the control). See Text S1 for post-mortem details. (**C**) Protocol to isolate slipped-DNAs from genomic DNA. Tissue DNA is isolated using a non-denaturing protocol (see Text S1). DNA is then digested to release the repeat-containing fragment at the DM1 locus from the rest of the genome (slipped-DNAs are not super-coil dependent), incubated with the anti-DNA junction antibody 2D3, pulled down using protein G beads, released from the beads, and then characterized. The *Bbs*I-(CTG)n-*Bam*HI restriction fragment size will vary depending upon the repeat size. NB, this image is best viewed directly on the original electronic image.

To verify that the anti-junction DNA antibody recognizes slipped-DNA structures with varying slip-out sizes and slip-out numbers, we assessed 2D3 binding to various slipped-DNAs using an electrophoretic mobility shift assay. Various slipped-DNA structures can form at trinucleotide repeats, and our lab has structurally characterized them in detail by electrophoresis, chemical and enzymatic probing, and electron microscopy [8], [9], [16]–[18]. While a slip-out does not translationally move along the repeat tract (slip or slide), multiple junction conformations occur and these are in dynamic equilibrium [8], [9], [16]–[18]. Previously, we demonstrated that 2D3 binds homoduplex slipped-DNAs of 50 CTG/CAG repeats on complementary strands as well as slipped intermediate heteroduplex DNAs (with an excess of 20 CAGs or 20 CTGs) [9]. These putative mutagenic intermediates may involve isolated slip-outs of various sizes, determined by the length difference between the two repeat-containing strands. Slipped-DNAs can also arise in tracts where the number of repeats between complementary strands does not differ, where each molecule contains multiple clustered short slip-outs [8], [9], [16]–[18]. For example each slipped molecule formed by (CTG)_50_•(CAG)_50_ contained 2–62 short slip-outs (1–31 on each strand), each composed of one to three repeat units [8], [9], [16]–[18]. To determine if 2D3 will recognize and bind to smaller slip-outs or clustered slip-outs, DNAs were made with isolated slip-outs of 1- or 3- or 20-excess repeat units of either the CTG or CAG strand, as well as a substrate with multiple clustered slip-outs on both strands along a tract of 50 repeats (Fig. 2A, see schematics). The antibody was incubated with each of these radioactively labeled DNAs, and resolved on polyacrylamide gels to visualize antibody-DNA complexes, evident as slower migrating than the protein-free DNA. Each of the substrates gave rise to slow-migrating species, where only the highest concentration led to partial shifting of the fully-duplexed control (leftmost lanes, see hollow arrowheads). These non-specific complexes were completely lost upon the addition of increasing amounts of competitor DNA (25-fold of non-specific competitor; linearized plasmid). This low-level non-specific binding, typical of many DNA-binding proteins/antibodies, has previously been observed to be readily competed-out [9], [11], [12]. In contrast, the lowest concentrations of 2D3 bound and shifted all of the slip-outs with an excess of 20 repeats (50×30 and 30×50) evident as several species (hollow and gray arrowheads). With the addition of competitor, these substrates remained completely shifted, but migrated faster (black arrowheads), indicating slip-out specific interaction. The 3- and 1-excess slip-outs with variable 2D3 concentrations also yielded shifted species, which were partially resistant to non-specific competition. The multiple shifted species may reflect different antibody-junction conformations [8], [9], [16]–[18], since each antibody can bind at either of the three junction angles, each of these could migrate differently and yield a broad and smeared appearance. The multiple species may also reflect different numbers of antibodies, as it was previously demonstrated that one to two 2D3 antibodies could bind a DNA-junction [9], [11], [12]. The clustered slip-outs were readily shifted by 2D3 and progressively shifted with increasing amounts of 2D3; indicative of increasing numbers of bound antibodies to the many slip-outs/molecule. We have previously shown this interaction to be resistant to competition [9], [11], [12]. Thus, 2D3 bound all sizes of slip-outs tested, with only very minor off-target binding to linear DNA that is eliminated after competition (Fig. 2A). 2D3 binds most effectively to larger slip-outs and clustered slip-outs. Importantly, the anti-DNA junction antibody did not induce the formation of slipped-DNA in fully-duplexed (CTG)_50_•(CAG)_50_ DNA (Fig. 2A, leftmost lanes), consistent with its inability to induce cruciform or hairpin extrusion [11], [12], [19]. Further control experiments with longer repeats are described below. 2D3 binding to slipped-DNAs was sensitive to proteinase K. Non-specific antibody-DNA binding was not seen with an isotype-matched anti-actin antibody control, which should not bind DNA (detailed in Supplementary Fig. S1). Thus, the anti-junction DNA antibody specifically recognizes a variety of slip-out sizes as well as DNA containing isolated and multiple clustered slip-outs; supporting the antibody as a useful tool to detect slipped-DNA structures.

To determine whether slipped-DNAs are present in a disease locus of patient tissues we developed a DNA-IP strategy (Fig. 2C). To be sure that we were not inducing slipped-DNA formation through manipulations (genomic isolation, IP etc.) we used several precautionary measures and performed a series of controls. Precautionary measures included preparing genomic DNAs under conditions that avoided DNA denaturation or shearing. Genomic DNAs were prepared from patient tissues under non-denaturing conditions to avoid inducing the formation of unusual DNA structures (see Fig. S1 and Text S1). Slipped-DNAs in CAG/CTG repeats cannot be induced by DNA supercoiling, and their biophysical stability does not depend upon supercoiling [8], [20]. Slipped DNAs can stably exist in linear unrestrained DNA [8]. To reduce DNA shear, enhance immunoprecipitation and eliminate the binding of 2D3 to supercoil-dependent DNA structures (cruciforms), genomic DNAs were restriction digested with *Bbs*I and *Bam*HI (which cut 180 bp upstream and 244 bp downstream of the CTG repeat, respectively, Fig. 2C), and then IP'd using 2D3 antibody and protein G agarose beads. While the 2D3 antibody does not induce cruciform or hairpin extrusion (see above) or slipped-DNAs in short fragments (less than 50 repeats) (Fig. 2A) it was important to assess its effect upon disease-relevant CTG expansions. Control experiments using *in vitro* prepared DNAs with (CTG)_500_•(CAG)_500_ showed that the 2D3 antibody did not induce slipped-DNAs into the expanded tracts (Fig. S2A, detailed in Text S1). Furthermore, we tested the possibility that structures might arise during genomic isolation in two ways; first, by adding the synthetically formed, fully-paired (CTG)_500_•(CAG)_500_ or (CTG)_50_•(CAG)_50_ DNAs to tissues to follow them through the genomic isolation protocol (Fig. S1), which did not induce slipped-DNA formation, and second, by assembling and disassembling nucleosomes on a supercoiled (CTG)_250_•(CAG)_250_ repeat plasmid, which again did not induce slipped-DNAs (Fig. S1). Thus, removal of chromatin from supercoiled repeats and the IP conditions used does not structurally alter the repeats from a fully-paired Watson-Crick duplex, consistent with previous reports [20]. Using these conditions and controls, we proceeded to assess the presence of slipped-DNA in patient tissues.

Towards determining whether the anti-junction antibody could recognize slip-outs that may have arisen at the expanded locus in human patient DNAs we first characterized the CTG tract length in a set of tissues from DM1 patients and a control non-DM1 individual by Southern blot (summarized in Fig. 2B, this LNA-Southern blot analyses have been previously published in [4]). A tissue-specific length variation of the expanded allele was evident in both patients used throughout this study, with many tissues showing a heterogeneous range of repeat sizes (Fig. 2B). Increased length heterogeneity within a tissue and larger expansions between tissues are indicative of high levels of active CTG instability, while limited length heterogeneity and shorter expansions reflect less instability [4]. The largest and most heterogeneous expansions were present in the most affected tissues (cortex, muscle and heart; indicated by broad repeat size ranges), while the cerebellum showed the shortest expansion with little or no length heterogeneity (as indicated by a relatively distinct sized fragment). Control non-DM1 samples showed only non-expanded alleles. These tissues were used throughout the study.

To determine whether 2D3 would recognize and bind to slipped-DNAs formed at the endogenous DM1 locus, we immunoprecipitated (IP'd) DNAs from various tissues, using the IP protocol (Fig. 2C) which would not be expected to either introduce or remove any pre-existing slipped-DNA structures. IP'd DNAs were subsequently characterized by various means, as described below.

**Figure 3:**
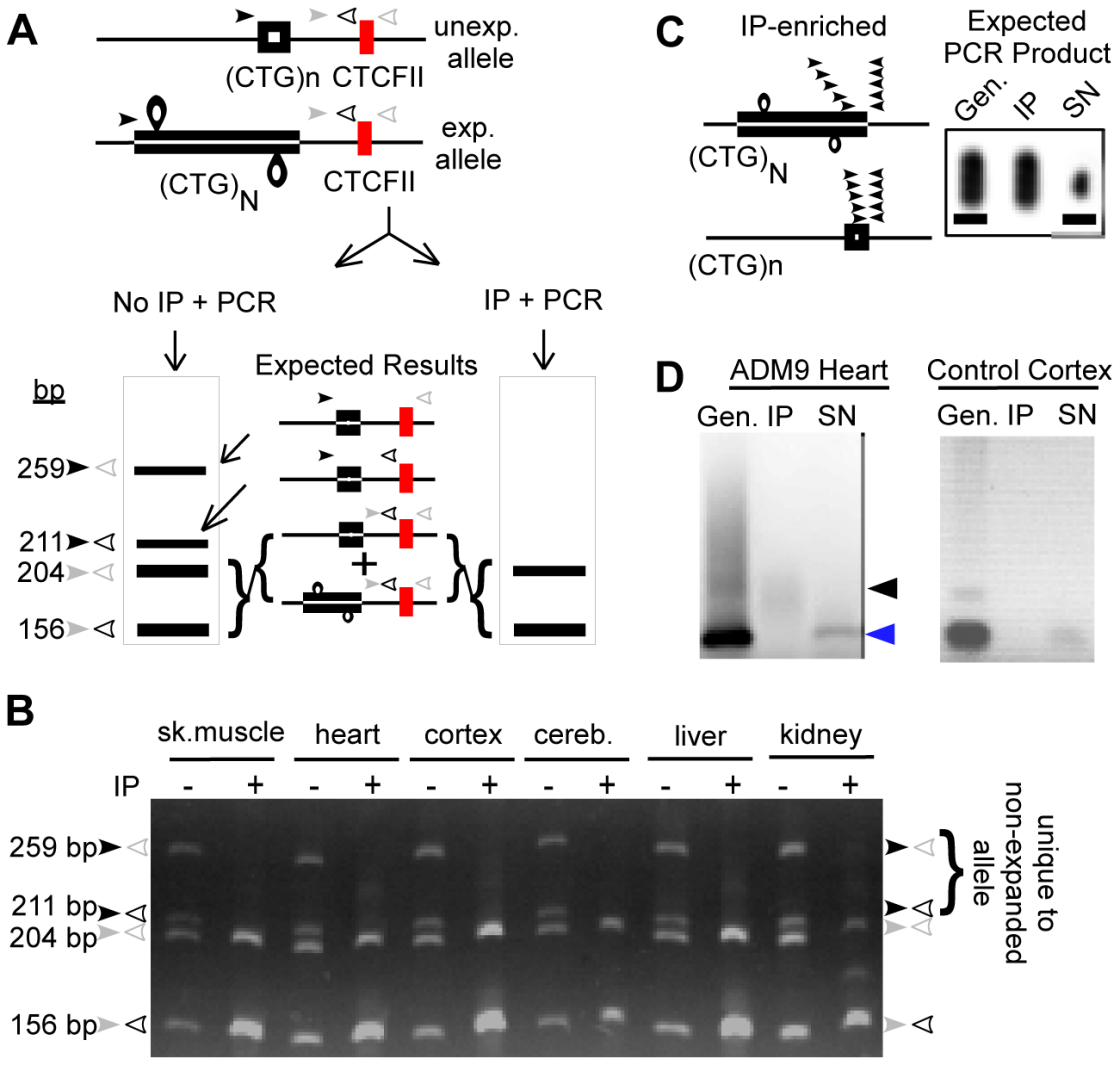
Immunoprecipitated DNA is enriched for the expanded DM1 allele. (**A**) Multiplex PCR protocol to determine the DM1 allele specificity of IP'd DNA, where “n” and “N” are the non-expanded and expanded alleles. Two primer pairs, indicated by arrow-heads are used in the same PCR reaction in order to differentiate between the expanded and non-expanded allele in genomic and IP'd DNA. Expected products are shown in the schematic gels for each case, sizes are based upon a non-expanded allele of (CTG)_4_. (**B**) Multiplex PCR analysis of ADM5 patient tissue DNAs shows only the lower two products in IP'd DNAs, indicating a strong enrichment of the expanded but not the non-expanded allele. DM1 individual, ADM5, has varying expanded repeat sizes between tissues – too large to be amplified across (Fig. 2B) and (CTG)_4_ in the non-expanded allele. Sizes of PCR products are indicated. The products in the IP lanes appear brighter because more DNA was loaded in these lanes in order to show that apparent loss of the larger PCR products that are unique to the non-expanded allele was not due to decreased sample loading. (**C**) Triplet-primed PCR protocol for IP'd DNAs (see text and Methods for full explanation of protocol). Briefly, an enrichment of the smeared PCR product (expanded allele) is expected over the smaller discrete product (non-expanded allele) after IP. (**D**) TP-PCR reveals predominantly the expanded allele in IP'd DNA (black arrowhead), and an absence of the non-expanded allele (blue arrowhead), confirming the specific immunoprecipitation of the expanded allele. The supernatant (SN) is depleted of the expanded but not the non-expanded allele. NB, this image is best viewed directly on the original electronic image. Neither Figure 3B nor Figure 3D are quantitative in nature; they are loaded in such a way that the differences between genomic and IP'd DNA are visually apparent.

Immunoprecipitation of slipped-DNA structures would be expected to enrich for the expanded disease-DM1 allele, as the non-expanded allele would not be expected to contain slipped-DNAs. Slipped-DNAs were in fact present on the expanded but not the non-expanded allele, as outlined: Because the expanded CTG tract in DM1 patient tissues is beyond the PCR amplifiable size range (1310–6550 repeats), we devised a multiplex PCR to distinguish the presence of the disease-allele from the non-disease allele in the immunoprecipitated DNAs. As outlined in Figure 3A, two sets of PCR primers amplifying adjacent overlapping regions of the DM1 locus were used in the same PCR reaction. The regions included the CTG repeat, and the downstream CTCF binding site. In the presence of both the expanded disease allele and the non-expanded allele, we expect four PCR products of distinct sizes. The largest two products would be unique to the non-expanded template derived from amplification across both the short CTG tract and the downstream CTCF site (259 bp), and the short CTG tract alone (211 bp). Amplification of the CTCF site only (204 bp) or the product in the overlapping region (156 bp) could arise from either allele. We did not expect either a PCR product from across the very large CTG expansion or from across the expansion and the adjacent CTCF site. Thus, when only the expanded CTG allele is present, we would not expect PCR products unique to the non-expanded allele (259 bp and 211 bp), whereas when both expanded and non-expanded alleles are present we would expect all four PCR products. This multiplex PCR assay was applied to 2D3-IP'd samples derived from various tissues of a DM1 patient (ADM5) with an expanded CTG tract of 1310–6550 repeats, and a non-expanded allele of (CTG)_4_ (Fig. 3B). Each genomic DNA prior to IP revealed all four multiplex PCR products, expected from both DM1 alleles (Fig. 3B, all lanes indicated by “−”). The IP'd DNAs revealed only the CTCF and overlapping PCR products (Fig. 3B, all lanes indicated by “+”). The absence of the upper two products, unique to the non-expanded allele provides strong support for the IP'd DNA to be enriched for the expanded DM1 allele, and deficient in the non-expanded allele. Multiplex PCR analysis of 2D3-IP'd DNAs from a non-DM1 control individual with DM1 alleles of (CTG)_5_ and (CTG)_25_ failed to detect any PCR products (Fig. S3A) – indicating that the 2D3 antibody could not detect any slipped-DNAs at either of the non-expanded alleles. The specificity of the 2D3-IP protocol to enrich for slipped-DNAs was supported by the inability to detect DNAs derived from a control region (lamin B2) that is devoid of unusual structure forming DNA sequence motifs (Fig. S3B). Thus, the ability to detect only those multiplex PCR products common to both the expanded and non-expanded alleles, but not those unique to the non-expanded allele, supports a specific enrichment of the expanded DM1 allele through anti-DNA junction IP. This strongly supports the presence of slipped-DNA in only the mutant expanded DM1 allele.

We used an independent direct method to determine the enrichment of the expanded DM1 allele in the IP'd material. Triplet-primed PCR (TP-PCR), which has been used as a DM1 diagnostic tool, allows for amplification of short stretches of a large expanded CTG tract [21]. TP-PCR uses a primer that hybridizes to the repeat and a primer that hybridizes downstream of the repeat (Fig. 3C). TP-PCR of the expanded CTG allele typically yields a heterogeneous range of CTG sizes in the PCR products, electrophoretically visible as a smear. This range of PCR products arises by PCR amplification of the repeat-specific primer hybridized randomly along the CTG expansion but relatively close to the flanking primer. TP-PCR across the non-expanded allele yields a distinct but shorter size range of PCR products, due to the limited locations to which the repeat-primer can hybridize (Fig. 3C). TP-PCR analysis of the DM1 patient genomic DNAs prior to IP revealed both the expected smear of products for the expanded allele and the distinct band for the non-expanded allele (Fig. 3D, “Gen.” lanes). However, TP-PCR of the IP'd sample revealed predominantly the longer range smeared products (“IP” lanes) derived from the expanded allele, while the DNA in the supernatant following IP from the same experiment revealed predominantly the shorter products derived from the non-expanded allele (“SN” lanes). These results directly support the enrichment of the expanded DM1 disease allele by the anti-DNA junction antibody, consistent with the interpretation that slipped-DNAs are present along the expanded allele.

**Figure 4:**
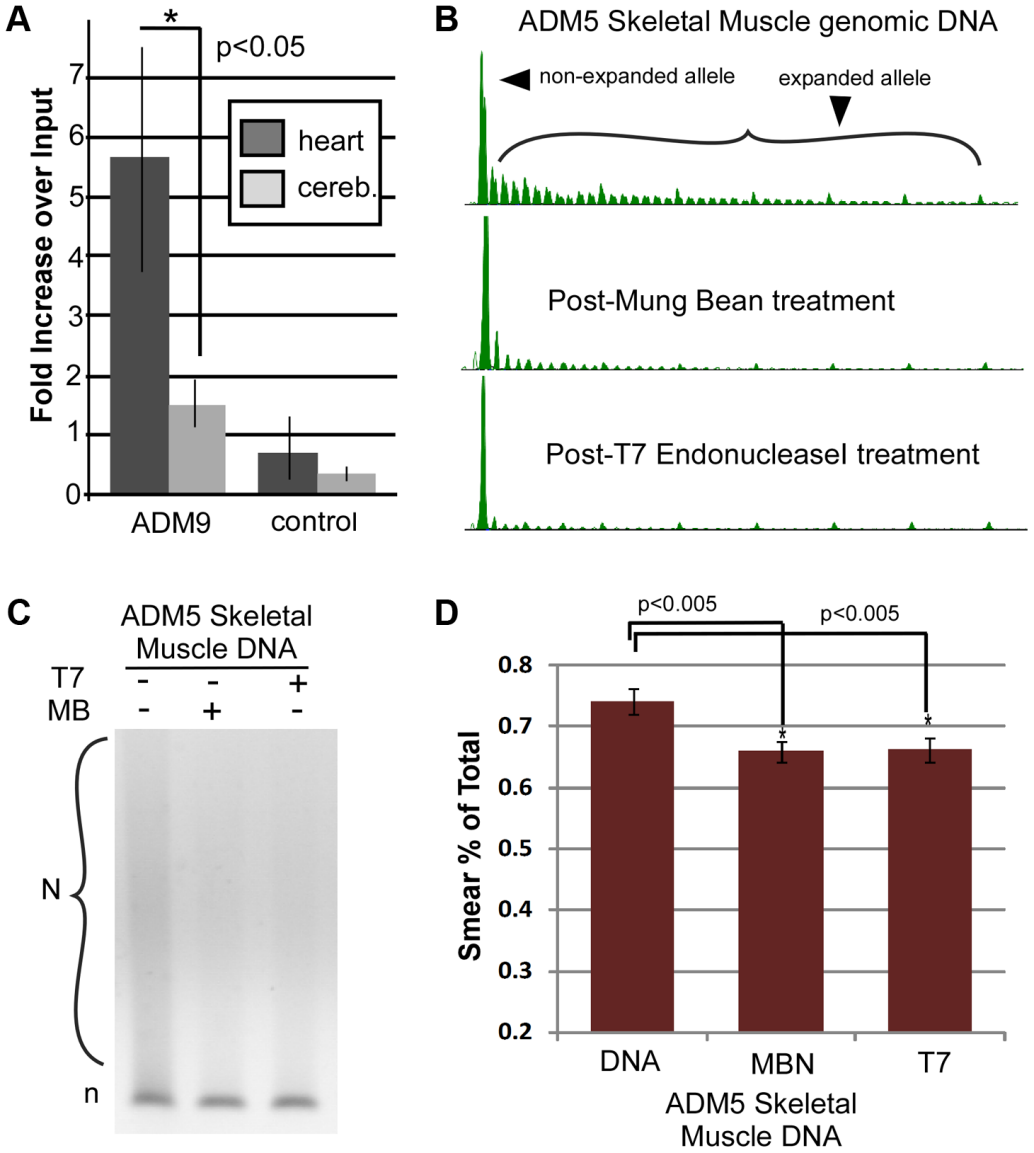
Quantitative and enzymatic analysis of patient DNA. (**A**) Quantitative competitive PCR revealed a significant increase in the amount IP'd/input from heart compared to cerebellum DNA of the same DM1 patient (unpaired two-tailed t-test, *p* = 0.03, n = at least 5 experimental replicates per treatment per tissue, on at least two genomic isolations). No significant difference was found between matched tissues of a non-DM1 individual. Details of quantitative competitive PCR and examples of the raw data are presented in Fig. S4. (**B**) Sensitivity of DM1 patient DNAs to structure-specific enzymes. For enzyme location specificity, see Fig. S4D TP-PCR analysis of samples +/−digestions assessed by GeneScan or (**C**) agarose electrophoresis, show decreased signal of the expanded allele after T7endoI or MBN digestion, with control DNA showing no difference. (**D**) Quantification of MBN and T7endoI digestion. Untreated heart DNA compared to MBN-treated, paired t-test, *p* = 0.0015, and compared to T7endoI-treated, paired t-test, *p* = 0.0015, n = at least 5 experimental replicates per treatment, on at least two genomic isolations. For analysis of additional tissues see Fig. S4. All errors bars indicate SEM, n = at least 5 experimental replicates per treatment per tissue, on at least two genomic isolations.

The levels of DM1 mutant alleles containing slipped-DNA might be expected to correlate with the levels of CTG instability, which varies between tissues of the same DM1 individual [4]. We quantified the amount of DM1 DNA being IP'd from two tissues from the same patient harboring high and low levels of CTG instability, indicated by the larger and heterogeneous CTG lengths in the heart (ranging from 4100–6000 repeats), and the more discrete and shorter length in the cerebellum (1100–1500 repeats, predominantly 1200 repeats), respectively (Fig. 2B). Quantification was accomplished using the highly accurate competitive quantitative PCR [22], [23] which involves the coamplification of a target DNA sample with known amounts of a cloned competitor DNA that shares most of the nucleotide sequence and primer sites with the target (detailed in Fig. S4A). Competitive PCR permits quantification of the absolute number of target molecules (a few representative examples are shown in Fig. S4B and S4C). Anti-DNA junction antibody IP'd DNA levels were significantly greater in the heart over the cerebellum (relative to input) of a DM1 individual (Fig. 4A), an enrichment that is consistent with a greater proportion of DM1 alleles with slipped-DNA present in tissues having greater levels of CTG instability. These findings indicate more DM1 alleles with at least one slipped-out, but cannot reveal the number of slip-outs per allele. DNA from a non-DM1 individual was not enriched for slipped-DNAs in either cerebellum or heart (Fig. 4A). These findings reveal a trend of increased levels of slipped-DNAs in tissues with higher levels of instability.

As a further test for the presence of slipped-DNAs in DM1 patient tissues, DNA was treated with enzymes that specifically digest features of slipped-DNA structures; a treatment that should eliminate slipped-DNAs. Mung bean nuclease (MBN) and T7 endonuclease I (T7endoI) have been shown to specifically cleave the single-stranded regions in slip-outs and across DNA-junctions of slipped-DNAs [8], [16] (Fig. S5D). Following enzyme digestions, DNAs were subjected to TP-PCR to assess the potential loss of the expanded alleles (Fig. 4B,C,D; additional examples in Fig. S5). If the expanded allele contains slipped-DNA, cleavage by these enzymes should decrease the amount of the smear representing the expanded allele. The amount of expanded repeat products in several DM1 patient tissues was significantly reduced following treatment with MBN or T7endoI (Fig. 4D, Fig. S5C). Non-DM1 control DNAs showed no difference after enzyme treatment (Fig. S5B,C). The significant reduction of the expanded allele by either structure-specific enzyme is consistent with a portion of the DM1 disease alleles being in the slipped-DNA conformation.

**Figure 5:**
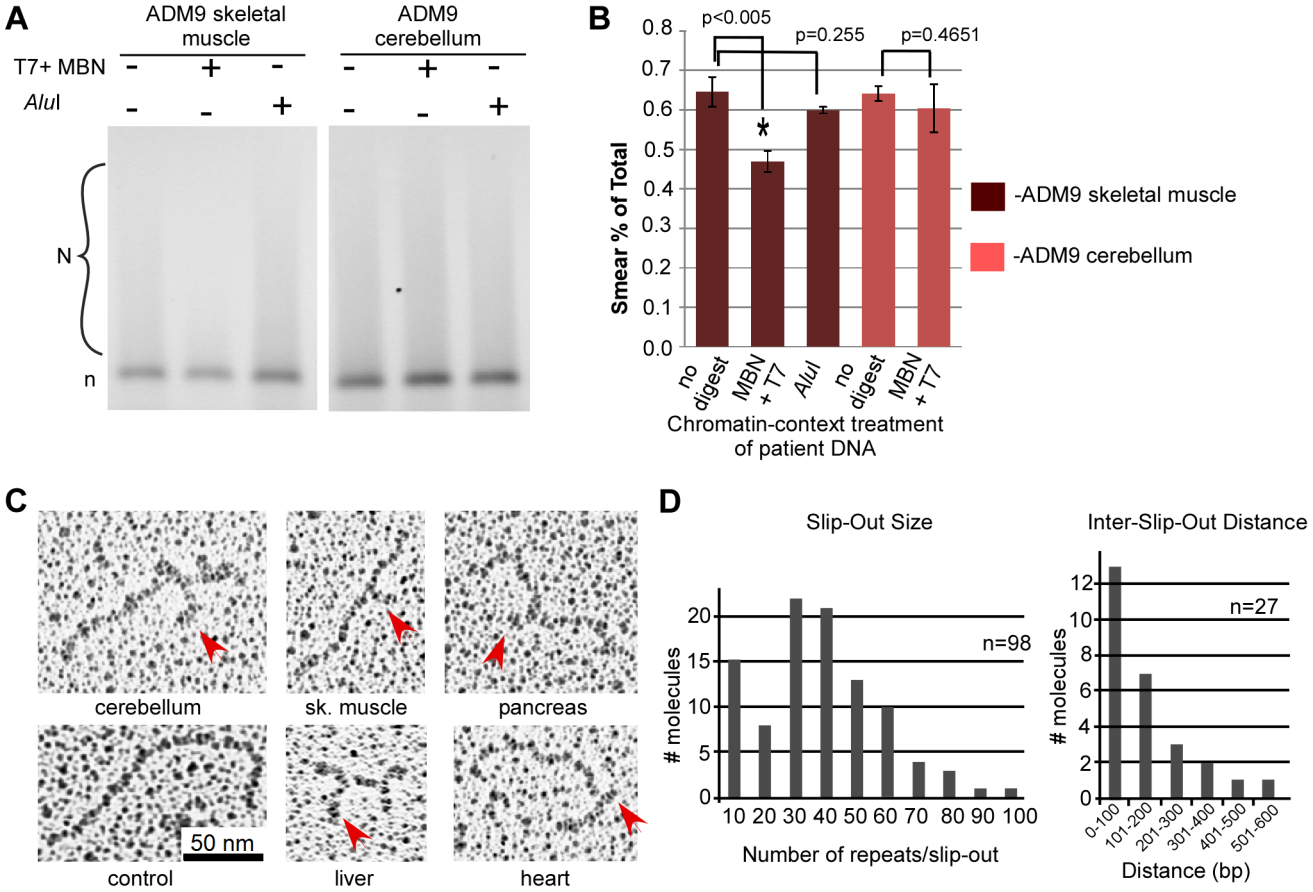
Analysis of slipped-DNA in native chromatin, and EM of IP'd DNA. (**A**) Tissues were treated in their native chromatin state with MBN and T7EndoI or *Alu*I enzymes, DNAs extracted and analyzed by TP-PCR. Agarose electrophoretic analysis of native-chromatin context digested DNA, run out after TP-PCR, showing a decrease in the expanded allele signal from patient muscle, but not cerebellum. See Figure S5A for representative GeneScan analyses of patient DNA treated in its native chromatin context with MBN and T7EndoI or *Alu*I enzyme (see Text S1 for Nuclease accessibility protocol). Also, see Figure S5C for a comparison of areas under each peak of the GeneScans before and after treatment. (**B**) The graph shows the significant difference in the reduction of the expanded allele after MBN and T7 treatment, compared to both untreated and *Alu*I treatment (p = 0.0038), n = 3 experiments. There is no significant difference between untreated and MBN+T7 treated ADM9 cerebellum digested in the native chromatin context. All error bars indicate SEM. (**C**) Electron microscopic imaging shows structured DNA. Electron microscopic (EM) images of immunoprecipitated DM1 DNAs and a control fully-duplexed DNA. IP'd DM1 tissue DNA shows multiple sized and clustered structures by EM. For EM analysis of additional tissue DNA as well as wider field views, see Supplementary Fig. S7. (**D**) Analysis of slip-out sizes and the distance between slip-outs on immunoprecipitated slipped DNAs. The size of the slip-outs presented a bimodal distribution ranging from 1–100 repeats with peaks at ∼30 and <10 repeats. Multiple slip-outs were clustered along a given DNA, with distances of <100 bp between slip-outs. NB, this image is best viewed directly on the original electronic image.

It was of interest to know if the slipped DNAs were present within tissues in the native chromatin context. Furthermore, the detection of slipped-DNAs directly on chromatinized DNA in tissues, where freeze and thawing of tissues does not affect chromatin packaging relative to fresh tissues [24], provides further evidence for the existence of slipped-DNAs prior to chromatin removal during DNA isolation in the above experiments. To assess the presence of slipped-DNAs in tissues, we used a slight modification of an established protocol of DNA nuclease accessibility assay on tissues, which assesses DNA in its native chromatin context [24] (see references in Text S1). Unusual DNA conformations have been detected in native chromatin through nuclease digestion [25]–[27]. For example, cruciform and stem-loops structures, similar to slipped-DNAs, were susceptible to nuclease digestion and found to stably reside in the inter-nucleosomal region [26], [27]. DM1 patient and control tissues were treated directly with either buffer only as a negative control, or both T7endonucleaseI and mung bean nuclease to test for digestion, or *Alu*I restriction enzyme to test for off-target digestion (Fig. 5A, B; Fig. S6). Following treatment, DNA was isolated and subjected to TP-PCR to assess the potential loss by digestion of the expanded alleles. There was a significant reduction in the expanded allele from patient ADM9 muscle tissue when treated with T7 and MBN nucleases (p = 0.0038), but not when treated with *Alu*I (p = 0.255). There was no significant reduction in either control cerebellum or heart, or patient cerebellum, after treatment with either T7 and MBN, or *Alu*I (Fig. 5A,B; Fig. S6). These results are consistent with the above results in that the tissue with greater CTG instability contains more slipped-DNAs, and control tissues do not. Thus, slipped-DNAs were present at the mutant DM1 locus, in the native chromatin in DM1 tissues and at levels that correlated with levels of instability.

Towards biophysically characterizing the slipped-DNAs, we visualized these by electron microscopy (EM). EM analysis of the IP'd slipped-DNAs revealed multiple bends, kinks, bulges, branched arms, and regions with increased thickness relative to control DNA (Fig. 5C, D, Fig. S7). A significantly increased number of molecules with visibly detectable slip-outs were identified in tissues showing high levels of CTG instability compared to the cerebellum, which showed the lowest level of instability (skeletal muscle vs. cerebellum, two-sided t-test; p = 0.005; pancreas vs. cerebellum, two-sided t-test; p = 0.0202; all unstable tissues (heart, liver, pancreas, cortex, skeletal muscle) vs. cerebellum; p = 0.0386; Fig. S6C). The variation of visibly detectable slip-outs between IP'd material of different tissues might indicate a slip-out size variation between tissues. The size of the slip-outs presented a bimodal distribution ranging from 1–100 repeats with peaks at ∼30 and <10 repeats (Fig. 5D). Multiple slip-outs were clustered along a given DNA, with distances of <100 bp between slip-outs (Fig. 5D). These features were also present in *in vitro* induced slipped-DNAs in synthetic (CTG)_800_•(CAG)_800_ (Fig. S7A) and are consistent with previous electron micrographs showing multiple short slip-outs in (CTG)•(CAG) tracts with 50–250 repeats [16], [17]. The IP'd DNAs were shorter than expected, possibly due to IP-induced DNA shearing of the very long repeat expansions that we studied, a phenomenon previously observed for DNAs enriched in single-strand nicks, gaps or DNAse I hypersensitive sites. An enrichment of such strand breaks may be expected for arrested repair of clustered slip-out lesions [28], a sensitivity to site fragility [29], and susceptibility of slipped-DNAs to double-strand breaks [30].

## Discussion

We report the isolation of slipped-DNAs present at an endogenous disease locus from various DM1 patient tissues. Our observations are supported by multiple independent, complementary experimental approaches. To better understand processes leading to disease-causing mutations, it is crucial to study these structures at specific genomic loci and in relevant patient tissues. This is particularly important for trinucleotide repeat mutations as these events are expansion-biased, arising by processes distinct from contractions, with instability patterns varying between disease loci, and between mitotic and non-mitotic tissues. An understanding of these DNA mutagenic intermediates occurring in patients should provide insight as to how they may be processed and lead to mutations, as model systems do not accurately reflect what is ongoing in affected individuals, especially on a tissue-specific level. Here, we reveal slipped-DNAs at the expanded CTG/CAG DM1 disease locus in tissues including the brain and heart. The brain in humans is essentially post-mitotic after birth [31], with both the cerebellum and cortex developing from very early on in embryogenesis and continuing up until the first years after birth [32], [33]. Similarly, the majority of cells in the heart are post-mitotic in nature, with cell division ceasing in cardiac myocytes soon after birth and the dividing cell population in adults being less than 1% of the total number of cells [34]–[36]. Since CTG expansions continue to expand at a greater rate following birth compared to prenatal expansion [4], our detection of slipped-DNAs in non-mitotic tissues supports that they arose in the absence of DNA replication. However, it is also possible that slipped-DNAs could form during early development and persist throughout the lifetime of an individual. Despite the similarity in timing of the post-mitotic nature of the frontal cortex, cerebellum and the heart, the cortex and heart showed significantly more slipped-DNA in patients compared to cerebellum, further supporting the hypothesis of the presence of tissue-specific instability factors. In DM1 and other CAG/CTG repeat diseases the tissue selectivity of repeat expansions correlates with the tissue selectivity of pathogenesis in the brain regions [37]–[41], the heart [42] and muscle [4], [43]. Our evidence at a patient locus from various tissues serves as the only evidence supporting the existence of slipped-DNAs in patients. Our observations of slipped-DNAs at a disease locus in post-mortem patient tissues, coupled with the report of hairpin DNAs at exogenously integrated repeats in cultured cell models [10] supports the formation of slipped-DNAs in patients. Importantly, we reveal a strong difference in the allelic levels of slipped-DNA between tissues of the same individual, and these levels correlate with the levels of instability in the tissues assessed.

**Figure 6:**
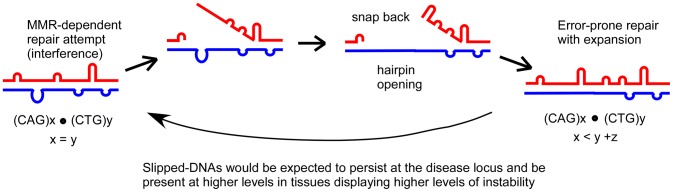
Proposed model for role of slipped-DNAs in repeat instability. Slipped homoduplex DNA that forms at a trinucleotide repeat may be a target of attempted repair. Interference by adjacent slip-outs may arrest repair, allowing for intrastrand slippage and the formation of a gap. When filled, this would result in an expansion in one of the strands, producing a heteroduplex as well as more slipped-DNA.

Surprisingly, slipped-DNAs are present as tandem clusters of multiple small slip-outs, with increased levels in DM1 patient tissues showing high repeat instability. This contrasts with the long-presumed concept that slipped-DNAs arise as isolated slip-outs on either strand of the duplex. That slipped-DNAs are clusters of short slip-outs gives new insight into the role they may play in the mutation process. The majority of analyses addressing the potential roles of slip-outs have centered upon the presumption that single isolated slip-outs may form transiently and be processed to fully-duplexed repeat-length mutations. Complementing our isolation of clustered slipped-DNA structures, it has been shown previously that clustered structures are poorly repaired compared to isolated single slip-outs [28]. The most striking implication of our findings is that slipped-DNAs do not appear to be merely transient in nature, as previously believed [44], but may be persistent *in vivo* products of instability, consistent with their poor ability to be repaired [28]. We propose that intrastrand slippage may occur after attempted repair events on slipped-DNAs become arrested due to adjacent slip-outs (Fig. 6). This shifts the repeats further out of register leaving a gap that, when filled, results in an increased number of repeats in that strand and producing slipped-DNAs with an excess of repeats on one or both strands. Reiterations of such events in the absence of proper repair would lead to instability, where products are slipped-DNAs.

Our IP protocol has identified slipped-DNAs at a CTG/CAG disease locus in patient tissues, structures that were not detected in appropriate controls (i.e., the antibody did not induce structure formation). While our findings are not definitive *in vivo* evidence, the fact that the allelic levels of slipped-DNAs varied between DM1 patient tissues, which showed varying levels of instability supports that these were formed in the patient. This concept is further supported by the detection of hairpins at integrated CTG/CAG repeats in cell models [10]. Different mutational paths involving slipped-DNAs may involve specific DNA repair pathways. For example, the mismatch repair complex MutSβ is required for CAG/CTG expansions [1]–[3]. Similarly, the levels and biophysical structure of slipped-DNAs forming at mono- and dinucleotide repeats may be distinct between tumors showing microsatellite instability compared to those that are microsatellite stable. Slipped-DNA identification and characterization could provide mechanistic and prognostic insights into medically important mutations in the many repeat-disease loci, hypermutable viruses, mitochondria, hypervariable genomic regions, and fragile sites [45]–[49] as it has done herein for DM1 repeat instability.

## Materials and Methods

Human tissues analyzed in this study are listed in Fig. 2B. Autopsy tissues from a non-affected individual (ADN1) were obtained snap-frozen from the National Disease Research Interchange. DNA extractions from human tissues were carried out under conditions to minimize DNA denaturation, to avoid inducing unusual DNA structure formation. Procedures were performed in low binding tubes, which avoid DNA denaturation. Complete protocol can be found in Text S1. Human-CTG repeat lengths were assessed by Southern blot using an LNA probe [DIG-labeled (CAG)_7_-5′-gcAgCagcAgCagCagcAgca-3′], as described previously [4]. Non-expanded CTG alleles were sized by sequencing of the products obtained after PCR amplification (forward primer 409, reverse primer 407). Slipped-DNA structures for bandshift were made as previously described [9] with minor changes (see Text S1). Individual structures and varying concentrations of antibody were run on a 4% (w/v) polyacrylamide gel in 1× TBE buffer at a constant 150 V for 1.5 hours. Following electrophoresis gels were dried and autoradiographed. Fully-duplexed (CTG)_50_•(CAG)_50_ were used to test for specificity of binding, with linearized p-Bluescript plasmid additionally being used as a competitor during antibody binding. DNA-IP of slipped-structures was carried out using the anti-DNA junction monoclonal antibody 2D3. 1 ug of genomic DNAs were restriction digested with *Bam*HI and *Bbs*I overnight at 37°C. 50 ul of hybridoma 2D3 culture supernatant containing ∼5 ug/ml immunoglobulin was diluted 1∶1 with PBS and added to the restriction digested patient DNA the following day and incubated on ice for one hour. 80 ul Protein G beads were prepared and resuspended in 250 ul 1× TE, which were then added to the antibody-DNA mixture and incubated on ice for 1 hour, mixing occasionally. The antibody/DNA/bead complex was washed free of unbound DNA and the affinity purified DNA was eluted using TE+2% SDS. DNA was further purified by phenol-chloroform-isoamyl alcohol extraction, followed by 100% ethanol precipitation. IP'd DNA samples were analyzed by electron microscopy essentially as described [16]. Multiplex PCR and TP-PCR protocol conditions are described in Text S1. For structure specificity experiments, patient DNA was digested with MBN or T7EndoI overnight and used for TP-PCR with either a fluorescently labeled primer after which the products were analyzed by GeneScan, or with a non-fluorescently labeled primer after which the products were run on a 1% agarose gel for visual analysis. DNAs digested within their native chromatin context were subjected to a modified nuclease accessibility protocol before digestion and DNA isolation (Text S1). EM and nucleosome assembly and disassembly were carried out as previously reported (Text S1, references 9 and 42, respectively).

## Supporting Information

Figure S1 2D3 antibody characterization, genomic DNA isolation and nucleosome analyses. (A) The anti-DNA junction antibody 2D3 does not induce structure in long repeat-containing linear DNA (B) Genomic isolation protocol does not induce formation of slipped structures in (CTG)50•(CAG)50 or (CTG)500•(CAG)500 linear DNA fragments (C) Removal of histones does not induce slipped-DNA formation in a supercoiled disease-length (CTG)250•(CAG)250 repeat-containing plasmid. (A) To test whether the 2D3 antibody may induce structure in repeat-containing DNAs during the immunoprecipiation procedure, the 2D3 antibody was incubated with linear DNA containing (CTG)_500_•(CAG)_500_. Because our IP is performed after digestion of genomic DNA with restriction enzymes, this control experiment was carried out with linear repeat-containing DNA. Following IP, both the IP'd material and the supernatant were electrophoresed on gels capable of resolving slipped-DNAs (4% polyacrylamide gel) and repeats were detected by Southern blot with a (CTG)15/(CAG)15 probe. As a marker of slipped-DNAs, which migrate slowly, the (CTG)_500_•(CAG)_500_ fragments were alkaline denatured and renatured to induce slipped DNAs (S-DNA) (6) and this was also loaded. No DNA was detected in the IP'd material, and the starting material was in the SN with the same electrophoretic migration of a fully-duplexed DNA, indicating that 2D3 did not induce slipped-DNA formation in the (CTG)_500_•(CAG)_500_ and failed to IP it. (B) To ensure the genomic DNA isolation protocol (see Text S1) does not induce slipped-DNAs in disease-length CTG repeats, a genomic DNA isolation was carried out with the addition of ^32^P end-labeled (CTG)50•(CAG)50 or (CTG)500•(CAG)500 linear DNA fragments as a traceable entity. These were added to tissues. After genomic isolation, the untreated (no IP) labeled DNAs (−) were run alongside the linear DNAs that had been through the IP isolation (+) on a 4% polyacrylamide gel at a constant 200 V. No altered migration was observed between the two linear DNAs, indicating a lack of altered structure. (C) To ensure that the removal of nucleosomes from supercoiled DNA during DNA isolation does not induce slipped-DNAs in disease-length CTG repeats, we performed a control experiment: histones were assembled into nucleosomes on supercoiled plasmids containing (CTG)_250_•(CAG)_250_ repeats and then subsequently removed. The length of (CTG)_250_•(CAG)_250_ was previously shown to preferentially bind nucleosomes [3]. Various concentrations of histones were used (1∶1, 1∶2, and 1∶4 (w∶w) DNA∶histones) as seen in lanes 2–4, after which the repeat containing fragment was released from the plasmid backbone using *Eco*RI and *Hin*dIII digestion. Lane 5 shows the same plasmid not previously assembled with histones and only digested with *Eco*RI and *Hin*dIII. Lane 6 shows a plasmid in which slipped-DNA was induced by alkaline denaturation and renaturation [8] and then the repeat-containing fragment released. No altered migration was observed in the repeat containing fragments that had histones assembled and disassembled. Removal of nucleosomes from supercoiled DNAs with (CTG)_250_•(CAG)_250_ did not lead to the induction of slipped-DNAs, regardless of nucleosome levels.
(PDF)

Figure S2 Binding of 2D3 to slipped-structures is antibody specific. To test whether the band-shifts observed in Fig. 2A occurred due to the specific binding of the anti-junction antibody, or as a result of a large amount of non-specific antibody binding to DNA, we incubated slipped substrates with 0 ng, 25 ng, and 250 ng of anti-actin antibody, matched for the isotype (IgG_1_) of 2D3 (A). An equivalent amount of each end-labeled substrate (by radioactive Cerenkov count, 4000 CPM) was incubated with various amounts of antibody in borate buffer (pH 7.6) for 30 minutes on ice. Reactions were electrophoresed on a 4% polyacrylamide gel at 200 V. Gels were dried and autoradiographed. No protein-DNA complexes were observed at any antibody concentration tested, arguing against a non-specific interaction of the anti-DNA junction antibody to the slipped-DNAs. FD = fully-duplexed. S = slipped homoduplex. SI = slipped intermediate heteroduplexes.
(PDF)

Figure S3 (A) Multiplex PCR of 2D3-IP'd material from a non-DM1 individual. (B) 2D3 antibody does not pull-down non-specific DNAs void of junction-forming sequences. (A) Multiplex PCR analysis of IP'd DNAs from a non-DM1 control individual with DM1 alleles of (CTG)_5_ and (CTG)_25_. Multiplex PCR reactions of genomic non-IP'd DNAs contain 6 products derived from the indicated primers, with four having the indicated repeat tract lengths from either of the non-expanded alleles. Importantly, the IP'd material from this non-DM1 individual did not show any PCR products indicating that the 2D3 antibody could not detect any slipped-DNAs at either of the non-expanded alleles. The primers used were identical to those used in Fig. 3. (B) The lamin-B2 region was used as a control locus for immunoprecipitation, as it is free of sequence motifs capable of assuming unusual DNA structures (there are no DNA junction-forming sequence motifs, such as slipped –DNAs, hairpins, cruciforms, Z-DNAs, triple-stranded DNAs, or quadruplex DNAs), and therefore should not be pulled down during IP with the 2D3 antibody. PCR amplification of the lamin-B2 locus from the 2D3-immunoprecipitated DNA (IP) did not yield detectable products. In contrast, the supernatant post-IP (SN), and the starting material (genomic) contained the lamin-B2 locus. Shown are representative PCR reaction products of DM1 patient ADM5 cerebellum DNAs run on a 4% polyacrylamide gel.
(PDF)

Figure S4 Competitive quantitative PCR. The immunoprecipitated material was quantified by the highly accurate competitive quantitative PCR [22], [23]. Calculation of the number of molecules in each IP'd sample was performed as outlined in panel A, and has been exhaustively described in two protocol papers [5], [22], [23], [50]. This method has been used to determine HIV viral loads [51], determine levels of oncogene amplification [52], map replication origins [5], [53], [54] and quantify ChIP'd proteins bound to chromosomal DNA [55]. We previously established this method for the DM1 locus to quantify the amounts of newly replicated DNA from different tissues [5]. More sensitive and reproducible than RT-PCR, quantitative competitive PCR involves the co-amplification of a known amount of input cloned competitor along with a set amount of IP'd material [22], [23]. A) Flowchart of quantitation. Preparation of competitor clone. The cloned competitor differs from the PCR target only by a small sequence insertion of around 50 bp to permit electrophoretic resolution of the competitor and template PCR products. In the left inset of panel A, the DM1 locus is shown (flanked by CTCF sites). The location at which IP'd DNA is being quantified along the locus (for example, the CTCF site used here) is PCR amplified, cloned, and into this clone a segment of DNA of roughly 50 bp is inserted between the primers. This competitor can now be amplified in the same reaction with similar efficiency as genomic DNA, but will be electrophoretically resolvable when run on a gel (running as the slower product). The cloned competitor is then calibrated using genomic DNA of the patient tissue from which IP'd DNA is to be analyzed, this calibration provides both a usable competitor range as well as a defined concentration of the extracted genomic DNA prior to IP, this concentration will be used as the starting pre-IP concentration. A known amount of these genomic samples were then subjected to 2D3-IP. The appropriate dilution of the IP'd material suitable for competitive PCR was then empirically determined. This IP'd material was then subjected to competitive PCR, where multiple reactions having the same amount of IP'd DNA and dilutions of known amounts of competitor (in numbers of molecules) were co-amplified, panel A lower inset. The number of molecules of the input IP'd material is accurately reflected by the numbers of input competitor molecules when the amount of competitor (C) PCR product is equivalent to the amount of target (IP'd DNA) PCR product (T). Competitive PCR was carried out in a Perkin-Elmer 4800 thermal cycler. Products were resolved by 4% polyacrylamide gel electrophoresis, stained with ethidium bromide and the band intensity of both competitor (C) and target (T) products quantified by densitometry on the gels (ImageQuant). The C/T ratio is plotted (y-axis) against the known number of molecules of input competitor (x-axis). The experimental points are fitted by a linear function. According to the equation describing this line, f(x) = mx+b, the number of competitor molecules corresponds to a C/T ratio = 1 is evaluated (the x value where f(x) = 1). This number is also the number of target 2D3-IP'd molecules initially present in the competitive PCR reaction. This amount (corrected for the dilution of IP'd material used) was correlated to the amount of starting pre-IP genomic equivalents. These relative amounts of 2D3-IP'd material from multiple independent assessments are graphed in Figure 4A. B & C) Representative quantitative competitive PCRs for tissues quantified for a given 2D3-IP of ADM9 heart and cerebellum as well as of from a control individual (independent immunoprecipitations with different genomic extractions differing in IP dilutions). These are only a few of many analyses. In each gel, the upper band is the PCR product of the competitor (C) and the lower band is the PCR product of the IP'd target (T) material of the specified tissue. The C/T ratios (below the gel) and numbers of input competitor templates (above the gel) are indicated for the reactions/lanes that approximate the 1∶1 C/T ratio (the exact 1∶1 ratio and exact numbers of molecules determined from the resulting linear equation, f(x) = mx+b = C/T, solving for x when C/T = 1 yields the number of input IP'd molecules). On the left of each gel image are the graphed densitometric C/T ratios versus known input amount of competitor (numbers of C molecules). Shown in the below the box is the number of IP'd molecules in the reaction where C/T = 1. D) The results from this set of IPs, where genomic isolations, 2D3-IP's and PCRs were performed in parallel, is graphed. The full data, derived from multiple independent genomic isolations and independent IPs, are summarized in Figure 4A.
(PDF)

Figure S5 DM1 patient DNAs are sensitive to structure specific enzymes at the DM1 locus. Mung bean nuclease (MBN) and T7 endonuclease I (T7endoI) recognize and digest specific features of slipped-DNAs. MBN is specific for single-stranded DNA including hairpin tips and loops of slip-outs, while T7endoI cleaves at the arms of three-way DNA junctions, common to slipped-DNA formed from CTG/CAG repeats [4], [17]. 10 Units of MBN or T7endoI were incubated with 200 ng DM1 patient genomic, un-IP'd DNA in NEB Buffer 2 (New England Biolabs) at 30°C for 30 minutes and 37°C for 30 minutes, respectively. After digestion, the DNA was subjected to TP-PCR (see Supplementary Table S1 for primers). Products of TP-PCR with a fluorescently labeled primer were analyzed by capillary electrophoresis, with scans shown in (A) where the green line plots the TP-PCR products. In (B) products of TP-PCR were run on a 1% agarose gel at a constant 100 V. Reduced amounts of the expanded repeat allele are visible in (A) as a reduced tail running towards the right of the scan, and (B) as a reduction in the PCR product smear. The reduction of the expanded allele was quantified by ImageQuant software as a percentage of the total amount of DNA in each lane, compared to untreated genomic DNA. ImageQuant quantifies pixel intensity in a selected region of the gel, corrected for background intensity. (C) There is a significant reduction in the expanded allele after treatment with both enzymes in both patients skeletal heart muscle (paired two-tailed t-test, ADM5 skeletal muscle genomic DNA vs MBN treatment, p = 0.0015, and vs. T7EndoI treatment, p = 0.0015; ADM9 skeletal muscle genomic DNA vs. MBN treatment, p = 0.003, and vs T7EndoI treatment, p = 0.0009). There was no significant difference after MBN or T7endoI treatment in non-DM1 individual DNA. (D) Specificity of MBN and T7EndoI on structured DNA. The smear above the “control” DNA lane is used as an additional control, as some of the smear may be PCR artifact due to slightly excess amounts of DNA being loaded into the PCR reaction. Anything quantified above that control band could be considered “background”. Anything beyond that in the other lanes is true expanded allele amplification.
(PDF)

Figure S6 DM1 patient DNAs are sensitive to structure-specific enzymes when treated while still in their native chromatin context. Patient and control tissues were subjected to digestion by structure specific enzymes (MBN and T7endoI) or a control enzyme (*Alu*I) within the native chromatin context (see Text S1 “Nuclear Accessibility Assay”). (A) Representative GeneScans of control cerebellum, ADM9 cerebellum, control heart and ADM9 skeletal muscle digested by the indicated enzymes. (B) Agarose electrophoretic analysis of the indicated tissue DNAs after digestion and TP-PCR. Statistical analyses comparing each enzyme digestion treatment against the mock-digestion treatment showed no significant differences, except between the ADM9 muscle MBN/T7 treatment compared to ADM9 muscle no treatment (p = 0.0038 two-sided t-test). (C) A comparison of the areas under the peak in ADM9 muscle treatments (untreated, MBN+T7, and ALUI), and ADM9 cerebellum treatments (untreated, MBN+T7). Each peak is represented as a percentage of the first peak, the first peak being the start of the normal and expanded repeats combined. Additionally, the percent reduction in area under the peak from treated to untreated is given. Peak 9 is the location at which the normal allele ends and only the expanded allele is being scanned.
(PDF)

Figure S7 Immunoprecipitated DNAs show structure by electron microscopy (EM). Electron microscopic analysis of slipped-DNAs. (A) As a positive control for slipped-DNA, *in vitro*-induced slipped-DNAs in the synthetic (CTG)800 repeat containing DNA fragments and its non-slipped variant were analyzed by EM. The fully-base paired (CTG)800 fragment appears with smooth contours (left panel), while the (CTG)800 fragments with induced slipped-DNAs appear thicker, with bends and kinked structures (right two panels). (B) The same multiple clustered short slip-outs were present in the immunoprecipitated DM1 patient DNAs (see also Fig. 5), consistent with previous EM results showing multiple bends in (CTG) repeat containing DNAs with induced slipped-DNAs [16]. (C) Quantification of slipped out molecules by tissue, as a percentage of total number of molecules seen in an EM field. Total number of molecules counted per tissue ranged from 6 to 32. A significantly increased number of molecules with slip-outs were identified in tissues showing high levels of CTG instability compared to the cerebellum, which showed the lowest level of instability (skeletal muscle vs. cerebellum, two-sided t-test; p = 0.005; pancreas vs. cerebellum, two-sided t-test; p = 0.0202; all unstable tissues (heart, liver, pancreas, cortex, skeletal muscle) vs. cerebellum; p = 0.0386).
(PDF)

Table S1 Primers used during study. This table details the sequences of the various primers used in this study.
(PDF)

Text S1 Supporting notes and methods. This text describes in detail attributes of the reagents, materials, tissues, and assays used in this study, including, the Anti-DNA Junction Antibody (2D3), electron microscopic Imaging of immunoprecipitated DNAs, human tissues, DNA extraction procedures, CTG repeat length analysis, in vitro slipped-DNA structure formation, band-shift assay, DNA-immunoprecipitation, polymerase chain reaction protocols, mung bean nuclease and T7EndonucleaseI digestion, nuclear accessibility protocol, nucleosome assembly and disassembly on an expanded repeat-containing plasmid, and supporting references.
(DOCX)
