# Supplementary figures and images for: Detection of Slipped-DNAs at the Trinucleotide Repeats of the Myotonic Dystrophy Type I Disease Locus in Patient Tissues
Source: PLoS Genet. 2013 Dec 19;9(12):e1003866. doi: 10.1371/journal.pgen.1003866 (PMC3868534; doi:10.1371/journal.pgen.1003866)

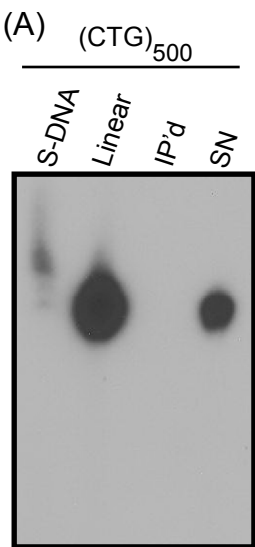

(B) Genomic Isolation

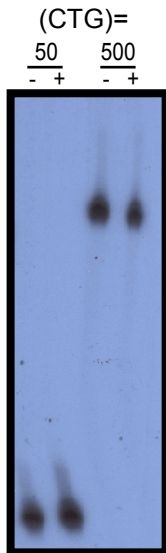

(C)

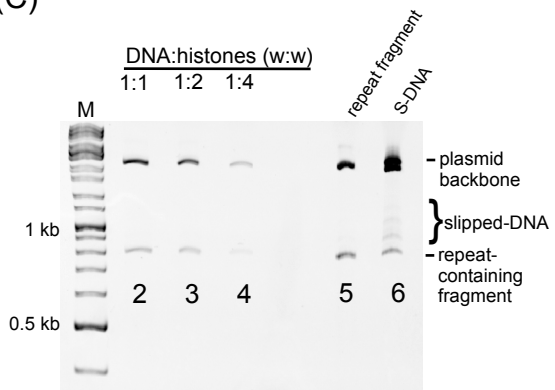

Supplement: Figure S1 — 2D3 antibody characterization, genomic DNA isolation and nucleosome analyses. (A) The anti-DNA junction antibody 2D3 does not induce structure in long repeat-containing linear DNA (B) Genomic isolation protocol does not induce formation of slipped structures in (CTG)50•(CAG)50 or (CTG)500•(CAG)500 linear DNA fragments (C) Removal of histones does not induce slipped-DNA formation in a supercoiled disease-length (CTG)250•(CAG)250 repeat-containing plasmid. (A) To test whether the 2D3 antibody may induce structure in repeat-containing DNAs during the immunoprecipiation procedure, the 2D3 antibody was incubated with linear DNA containing (CTG)500•(CAG)500. Because our IP is performed after digestion of genomic DNA with restriction enzymes, this control experiment was carried out with linear repeat-containing DNA. Following IP, both the IP'd material and the supernatant were electrophoresed on gels capable of resolving slipped-DNAs (4% polyacrylamide gel) and repeats were detected by Southern blot with a (CTG)15/(CAG)15 probe. As a marker of slipped-DNAs, which migrate slowly, the (CTG)500•(CAG)500 fragments were alkaline denatured and renatured to induce slipped DNAs (S-DNA) (6) and this was also loaded. No DNA was detected in the IP'd material, and the starting material was in the SN with the same electrophoretic migration of a fully-duplexed DNA, indicating that 2D3 did not induce slipped-DNA formation in the (CTG)500•(CAG)500 and failed to IP it. (B) To ensure the genomic DNA isolation protocol (see Text S1) does not induce slipped-DNAs in disease-length CTG repeats, a genomic DNA isolation was carried out with the addition of 32P end-labeled (CTG)50•(CAG)50 or (CTG)500•(CAG)500 linear DNA fragments as a traceable entity. These were added to tissues. After genomic isolation, the untreated (no IP) labeled DNAs (−) were run alongside the linear DNAs that had been through the IP isolation (+) on a 4% polyacrylamide gel at a constant 200 V. No altered migration was obser [file pgen.1003866.s001.pdf]

(A)

|         |           |          |           |           |           |           |
|---------|-----------|----------|-----------|-----------|-----------|-----------|
|         | <u>FD</u> | <u>S</u> | <u>SI</u> | <u>SI</u> | <u>SI</u> | <u>SI</u> |
| (CTG)n= | 50        | 50       | 47        | 50        | 30        | 50        |
| (CAG)n= | 50        | 50       | 50        | 47        | 50        | 30        |

Anti-actin Ab - 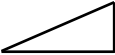 - 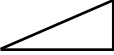 - 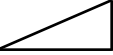 - 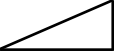 - 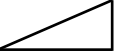 - 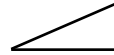

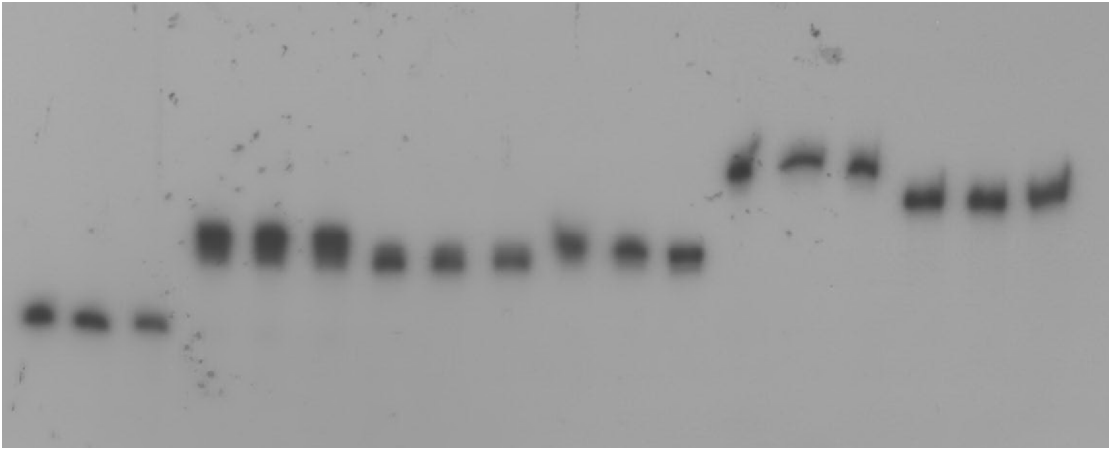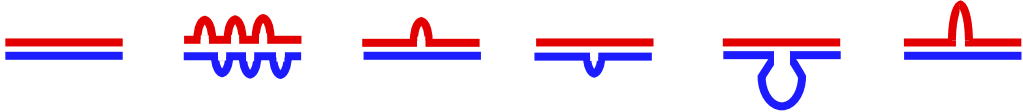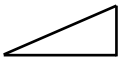

0 ng Ab  
25 ng Ab  
250 ng Ab

Supplement: Figure S2 — Binding of 2D3 to slipped-structures is antibody specific. To test whether the band-shifts observed in Fig. 2A occurred due to the specific binding of the anti-junction antibody, or as a result of a large amount of non-specific antibody binding to DNA, we incubated slipped substrates with 0 ng, 25 ng, and 250 ng of anti-actin antibody, matched for the isotype (IgG1) of 2D3 (A). An equivalent amount of each end-labeled substrate (by radioactive Cerenkov count, 4000 CPM) was incubated with various amounts of antibody in borate buffer (pH 7.6) for 30 minutes on ice. Reactions were electrophoresed on a 4% polyacrylamide gel at 200 V. Gels were dried and autoradiographed. No protein-DNA complexes were observed at any antibody concentration tested, arguing against a non-specific interaction of the anti-DNA junction antibody to the slipped-DNAs. FD = fully-duplexed. S = slipped homoduplex. SI = slipped intermediate heteroduplexes. (PDF) [file pgen.1003866.s002.pdf]

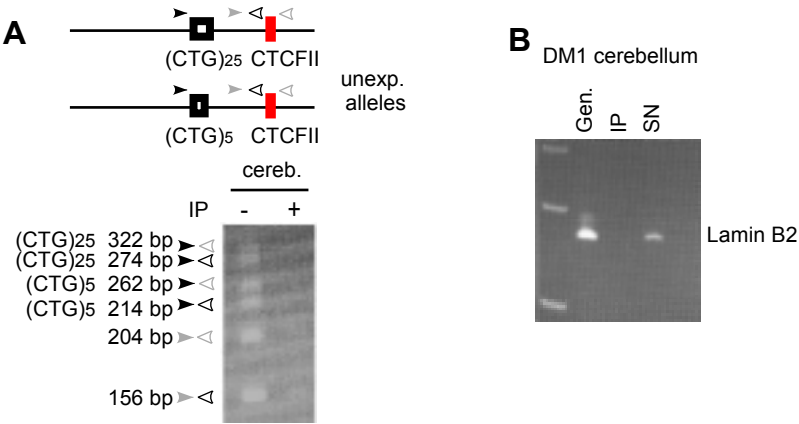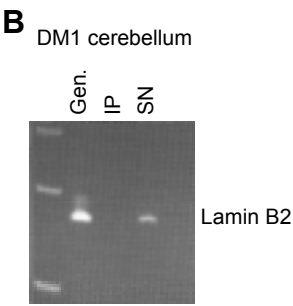

Supplement: Figure S3 — (A) Multiplex PCR of 2D3-IP'd material from a non-DM1 individual. (B) 2D3 antibody does not pull-down non-specific DNAs void of junction-forming sequences. (A) Multiplex PCR analysis of IP'd DNAs from a non-DM1 control individual with DM1 alleles of (CTG)5 and (CTG)25. Multiplex PCR reactions of genomic non-IP'd DNAs contain 6 products derived from the indicated primers, with four having the indicated repeat tract lengths from either of the non-expanded alleles. Importantly, the IP'd material from this non-DM1 individual did not show any PCR products indicating that the 2D3 antibody could not detect any slipped-DNAs at either of the non-expanded alleles. The primers used were identical to those used in Fig. 3. (B) The lamin-B2 region was used as a control locus for immunoprecipitation, as it is free of sequence motifs capable of assuming unusual DNA structures (there are no DNA junction-forming sequence motifs, such as slipped –DNAs, hairpins, cruciforms, Z-DNAs, triple-stranded DNAs, or quadruplex DNAs), and therefore should not be pulled down during IP with the 2D3 antibody. PCR amplification of the lamin-B2 locus from the 2D3-immunoprecipitated DNA (IP) did not yield detectable products. In contrast, the supernatant post-IP (SN), and the starting material (genomic) contained the lamin-B2 locus. Shown are representative PCR reaction products of DM1 patient ADM5 cerebellum DNAs run on a 4% polyacrylamide gel. (PDF) [file pgen.1003866.s003.pdf]

(A)

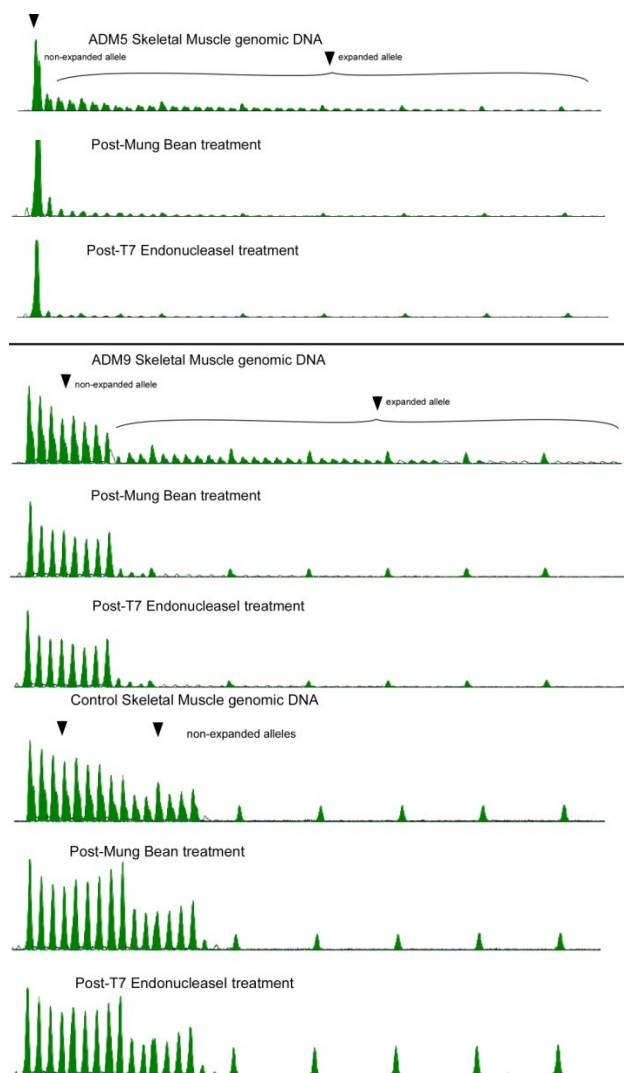

(B)

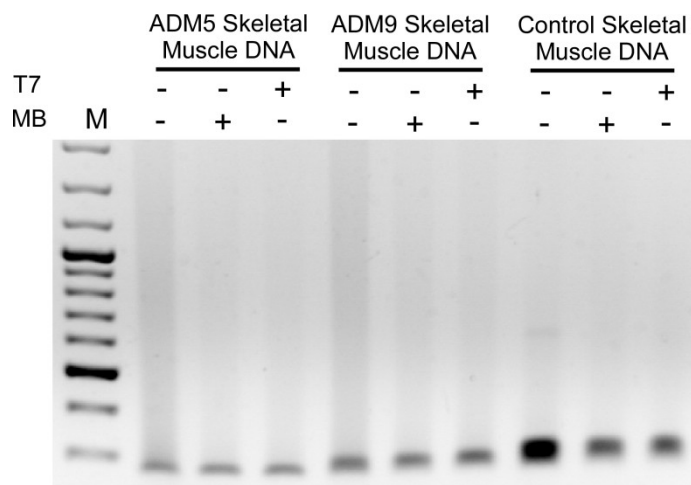

(C)

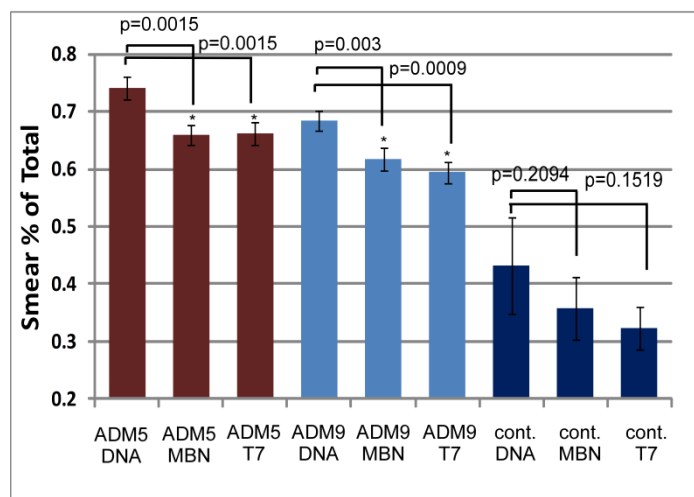

(D)

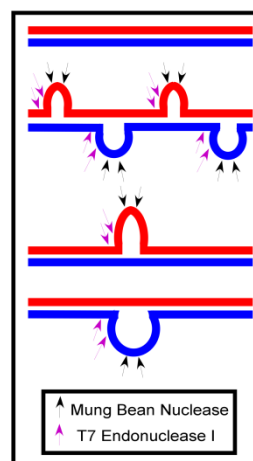

Supplement: Figure S5 — DM1 patient DNAs are sensitive to structure specific enzymes at the DM1 locus. Mung bean nuclease (MBN) and T7 endonuclease I (T7endoI) recognize and digest specific features of slipped-DNAs. MBN is specific for single-stranded DNA including hairpin tips and loops of slip-outs, while T7endoI cleaves at the arms of three-way DNA junctions, common to slipped-DNA formed from CTG/CAG repeats [4], [17]. 10 Units of MBN or T7endoI were incubated with 200 ng DM1 patient genomic, un-IP'd DNA in NEB Buffer 2 (New England Biolabs) at 30°C for 30 minutes and 37°C for 30 minutes, respectively. After digestion, the DNA was subjected to TP-PCR (see Supplementary Table S1 for primers). Products of TP-PCR with a fluorescently labeled primer were analyzed by capillary electrophoresis, with scans shown in (A) where the green line plots the TP-PCR products. In (B) products of TP-PCR were run on a 1% agarose gel at a constant 100 V. Reduced amounts of the expanded repeat allele are visible in (A) as a reduced tail running towards the right of the scan, and (B) as a reduction in the PCR product smear. The reduction of the expanded allele was quantified by ImageQuant software as a percentage of the total amount of DNA in each lane, compared to untreated genomic DNA. ImageQuant quantifies pixel intensity in a selected region of the gel, corrected for background intensity. (C) There is a significant reduction in the expanded allele after treatment with both enzymes in both patients skeletal heart muscle (paired two-tailed t-test, ADM5 skeletal muscle genomic DNA vs MBN treatment, p = 0.0015, and vs. T7EndoI treatment, p = 0.0015; ADM9 skeletal muscle genomic DNA vs. MBN treatment, p = 0.003, and vs T7EndoI treatment, p = 0.0009). There was no significant difference after MBN or T7endoI treatment in non-DM1 individual DNA. (D) Specificity of MBN and T7EndoI on structured DNA. The smear above the “control” DNA lane is used as an additional control, as some of the smear may be PCR artifact du [file pgen.1003866.s005.pdf]
