# Supplementary material for: Detection of Slipped-DNAs at the Trinucleotide Repeats of the Myotonic Dystrophy Type I Disease Locus in Patient Tissues
Source: PLoS Genet. 2013 Dec 19;9(12):e1003866. doi: 10.1371/journal.pgen.1003866 (PMC3868534; doi:10.1371/journal.pgen.1003866)
Supplement: Figure S4 — Competitive quantitative PCR. The immunoprecipitated material was quantified by the highly accurate competitive quantitative PCR [22], [23]. Calculation of the number of molecules in each IP'd sample was performed as outlined in panel A, and has been exhaustively described in two protocol papers [5], [22], [23], [50]. This method has been used to determine HIV viral loads [51], determine levels of oncogene amplification [52], map replication origins [5], [53], [54] and quantify ChIP'd proteins bound to chromosomal DNA [55]. We previously established this method for the DM1 locus to quantify the amounts of newly replicated DNA from different tissues [5]. More sensitive and reproducible than RT-PCR, quantitative competitive PCR involves the co-amplification of a known amount of input cloned competitor along with a set amount of IP'd material [22], [23]. A) Flowchart of quantitation. Preparation of competitor clone. The cloned competitor differs from the PCR target only by a small sequence insertion of around 50 bp to permit electrophoretic resolution of the competitor and template PCR products. In the left inset of panel A, the DM1 locus is shown (flanked by CTCF sites). The location at which IP'd DNA is being quantified along the locus (for example, the CTCF site used here) is PCR amplified, cloned, and into this clone a segment of DNA of roughly 50 bp is inserted between the primers. This competitor can now be amplified in the same reaction with similar efficiency as genomic DNA, but will be electrophoretically resolvable when run on a gel (running as the slower product). The cloned competitor is then calibrated using genomic DNA of the patient tissue from which IP'd DNA is to be analyzed, this calibration provides both a usable competitor range as well as a defined concentration of the extracted genomic DNA prior to IP, this concentration will be used as the starting pre-IP concentration. A known amount of these genomic samples were then subjected to 2D3-IP. The ap [file pgen.1003866.s004.pdf]

Fig. S4A

Prepare competitors

↓

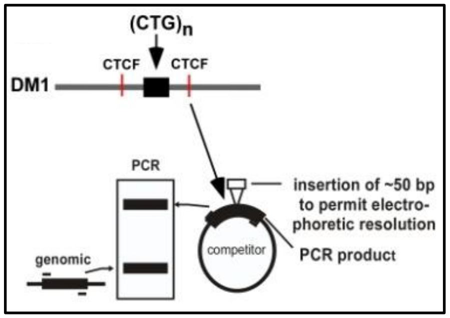

Isolate genomic DNA from tissue

↓

Determine pre-IP DNA concentration

↓

2D3 – IP
(determine dilutions of pre-IP and post-IP needed)

↓

Competitive PCR using a range of known amounts of competitor and a fixed concentration of IP'd DNA

↓

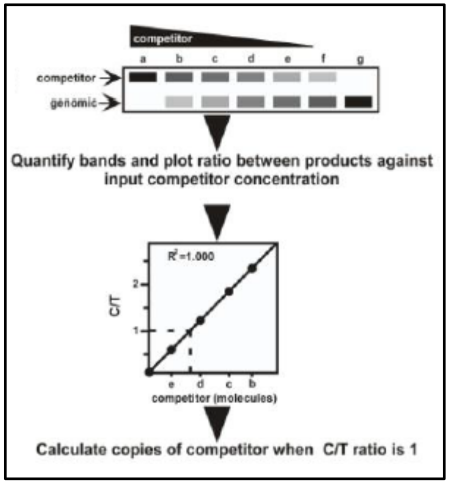

Electrophorese, stain and perform densitometric analysis of competitor and target products

↓

Plot C/T vs number of input Competitor molecules obtain linear function $f(x) = mx + b$

↓

Determine "x" when $f(x) = C/T = 1$
(x = number of input target molecules)

↓

Calculate number of 2D3-IP molecules (correcting for IP dilution) relative to pre-IP DNA genomic equivalents

↓

Summarize each IP set (Fig. S4F) as fold increase over input (see Fig 4A)

Fig. S4B

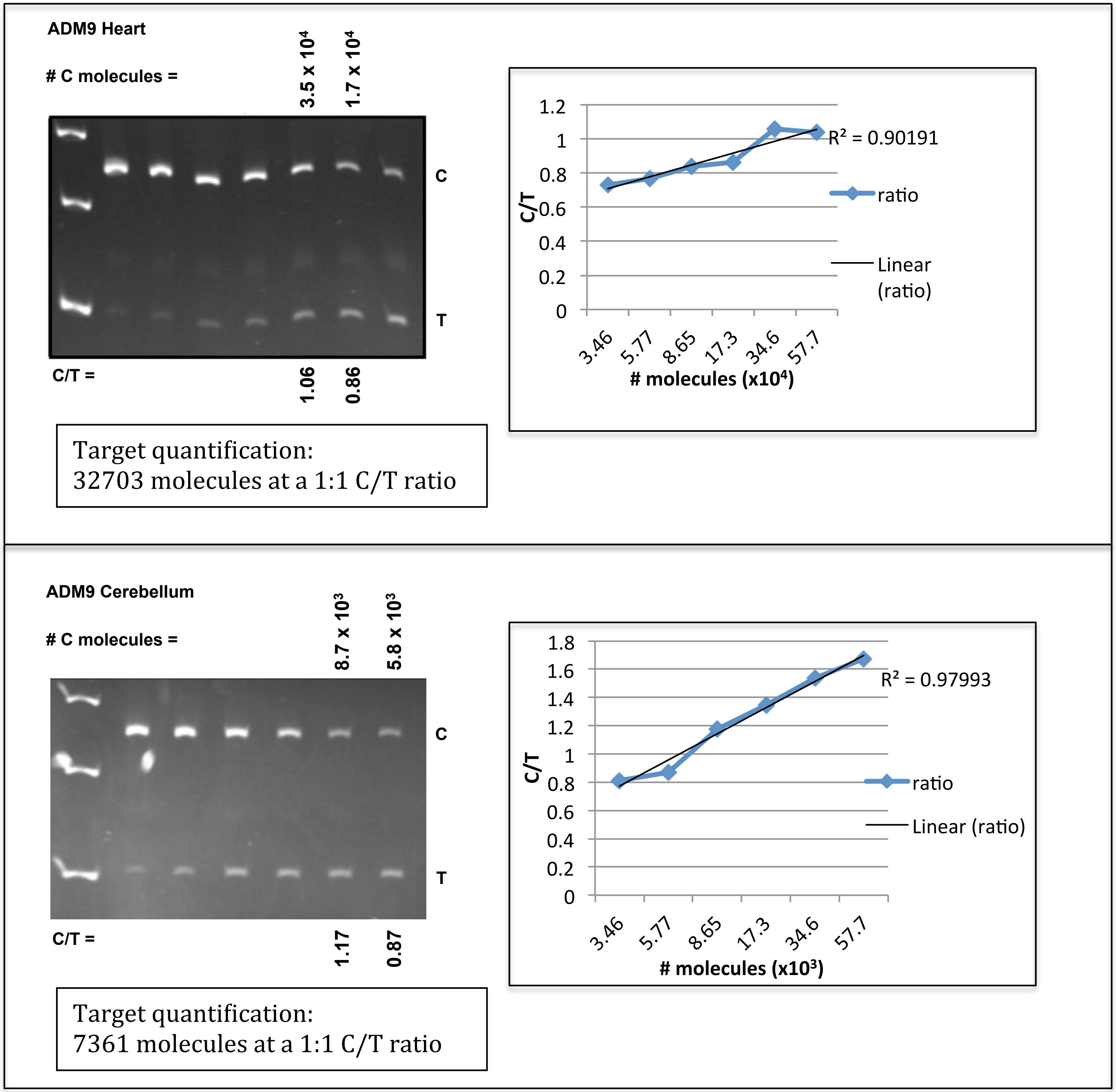
ADM9 Heart
C molecules =
3.5 x 10⁴
1.7 x 10⁴
C
T
C/T =
1.06
0.86
Target quantification:
32703 molecules at a 1:1 C/T ratio
1.2
1
0.8
0.6
0.4
0.2
0
C/T
3.46
5.77
8.65
17.3
34.6
57.7
# molecules (x10⁴)
R² = 0.90191
ratio
Linear (ratio)
ADM9 Cerebellum
C molecules =
8.7 x 10³
5.8 x 10³
C
T
C/T =
1.17
0.87
Target quantification:
7361 molecules at a 1:1 C/T ratio
1.8
1.6
1.4
1.2
1
0.8
0.6
0.4
0.2
0
C/T
3.46
5.77
8.65
17.3
34.6
57.7
# molecules (x10³)
R² = 0.97993
ratio
Linear (ratio)

Fig. S4C

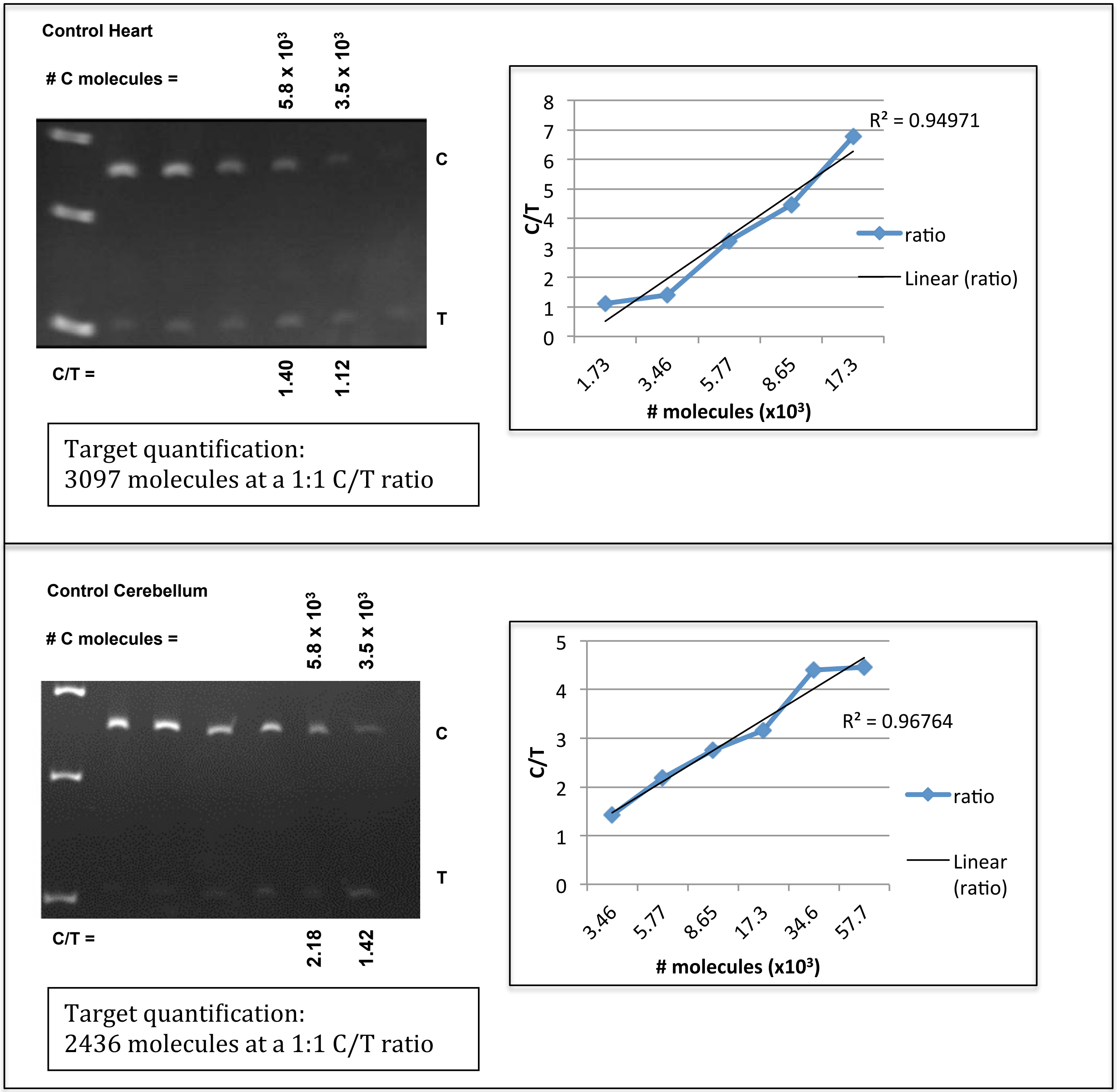
Control Heart
C molecules =
5.8 x 10³
3.5 x 10³
C
T
C/T =
1.40
1.12
Target quantification:
3097 molecules at a 1:1 C/T ratio
R² = 0.94971
C/T
ratio
Linear (ratio)
1.73
3.46
5.77
8.65
17.3
molecules (x10³)
Control Cerebellum
C molecules =
5.8 x 10³
3.5 x 10³
C
T
C/T =
2.18
1.42
Target quantification:
2436 molecules at a 1:1 C/T ratio
R² = 0.96764
C/T
ratio
Linear (ratio)
3.46
5.77
8.65
17.3
34.6
57.7
molecules (x10³)

Fig. S4D

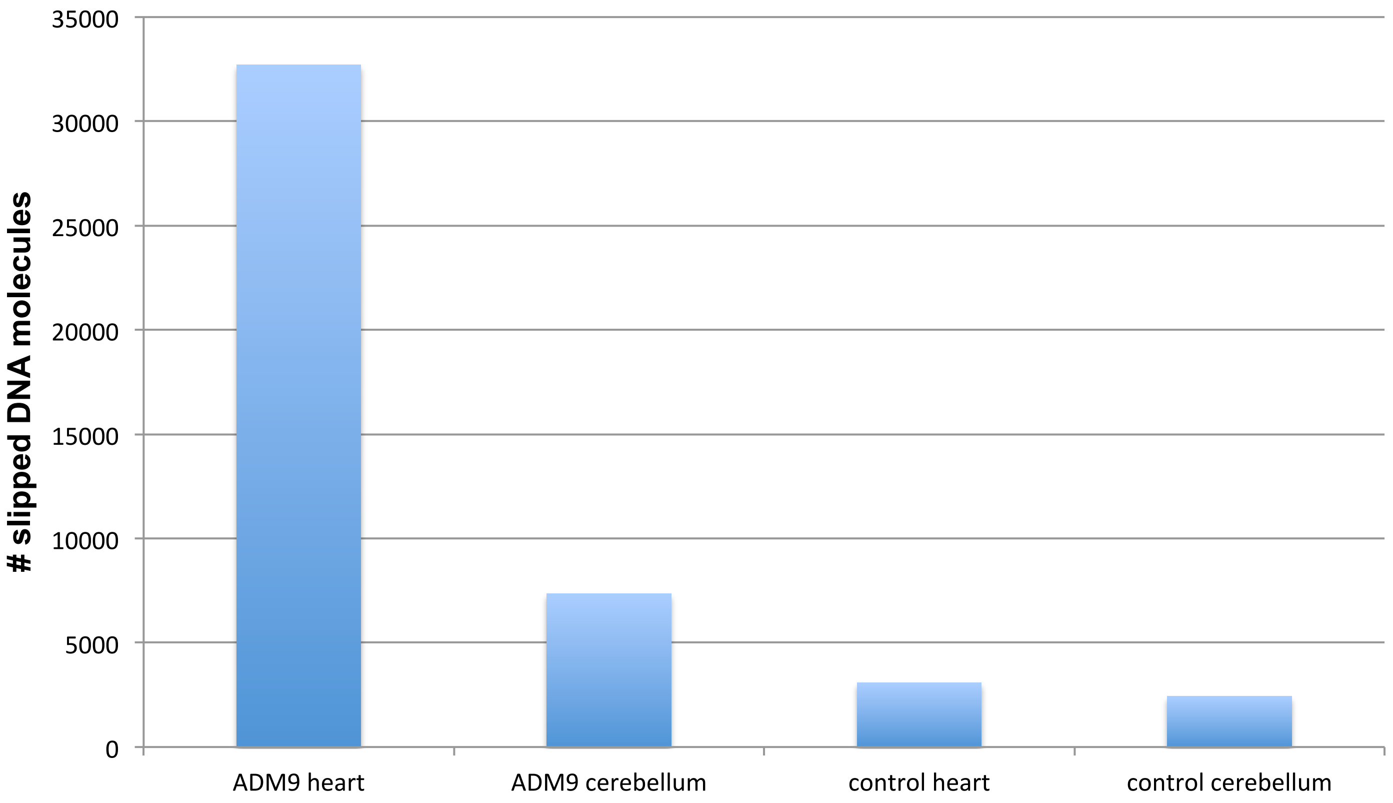

35000
30000
25000
20000
15000
10000
5000
0
slipped DNA molecules
ADM9 heart
ADM9 cerebellum
control heart
control cerebellum
