# Supplementary material for: Detection of Slipped-DNAs at the Trinucleotide Repeats of the Myotonic Dystrophy Type I Disease Locus in Patient Tissues
Source: PLoS Genet. 2013 Dec 19;9(12):e1003866. doi: 10.1371/journal.pgen.1003866 (PMC3868534; doi:10.1371/journal.pgen.1003866)

(A)

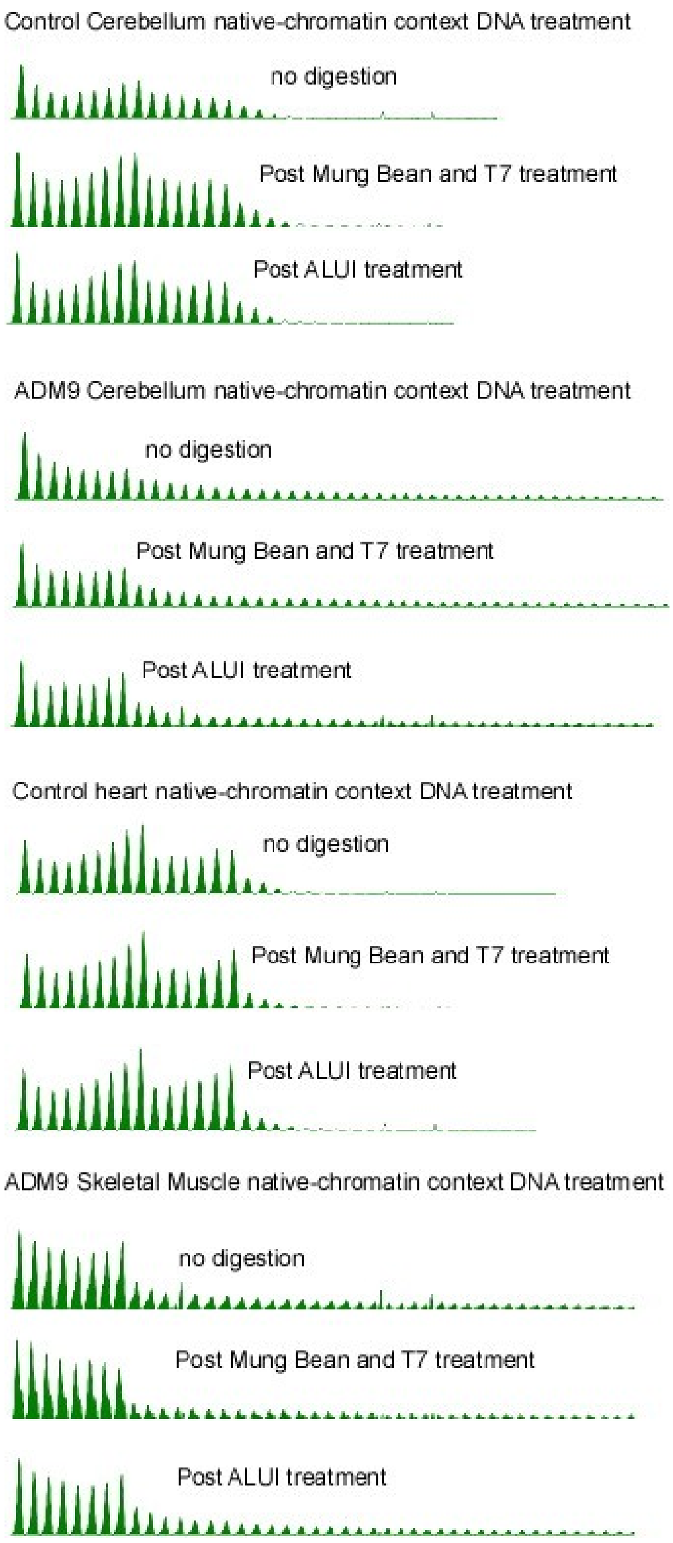

(B)

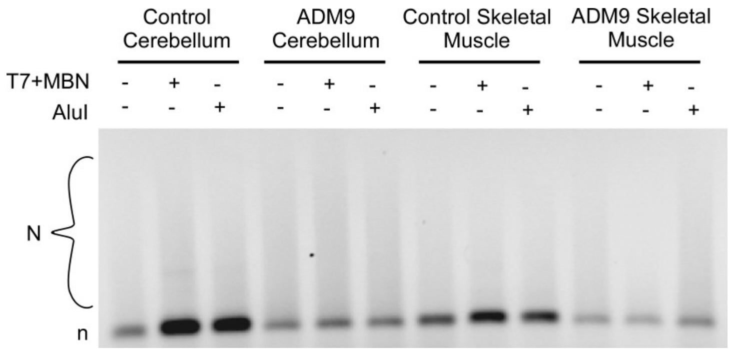

(C)

Areas Under the Peak of ADM9 Muscle (as a % of the first peak in the normal allele)				ADM9 Cerebellum	
Peak Comparison	Untreated	MBN + T7	AluI	Untreated	MBN + T7
**Expanded allele**					
CTG 1 vs CTG 9	35	18 / 48	38	32	35 / -9
CTG 1 vs CTG 10	27	15 / 44	31	30	30 / 0
CTG 1 vs CTG 11	22	13 / 41	25	26	25 / 4
CTG 1 vs CTG 12	24	11 / 54	21	22	21 / 5
CTG 1 vs CTG 13	19	13 / 32	24	23	22 / 4
CTG 1 vs CTG 14	19	11 / 42	22	20	19 / 5
CTG 1 vs CTG 15	19	11 / 42	21	20	20 / 0
CTG 1 vs CTG 16	15	9 / 40	17	19	18 / 5
CTG 1 vs CTG 17	15	9 / 40	17	17	17 / 0
CTG 1 vs CTG 18	13	11 / 15	15	16	15 / 6
CTG 1 vs CTG 19	14	11 / 21	15	15	15 / 0
CTG 1 vs CTG 20	13	8 / 38	14	15	15 / 0

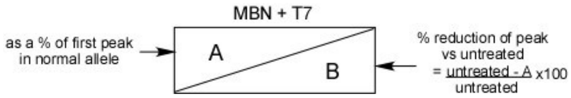

Supplement: Figure S6 — DM1 patient DNAs are sensitive to structure-specific enzymes when treated while still in their native chromatin context. Patient and control tissues were subjected to digestion by structure specific enzymes (MBN and T7endoI) or a control enzyme (AluI) within the native chromatin context (see Text S1 “Nuclear Accessibility Assay”). (A) Representative GeneScans of control cerebellum, ADM9 cerebellum, control heart and ADM9 skeletal muscle digested by the indicated enzymes. (B) Agarose electrophoretic analysis of the indicated tissue DNAs after digestion and TP-PCR. Statistical analyses comparing each enzyme digestion treatment against the mock-digestion treatment showed no significant differences, except between the ADM9 muscle MBN/T7 treatment compared to ADM9 muscle no treatment (p = 0.0038 two-sided t-test). (C) A comparison of the areas under the peak in ADM9 muscle treatments (untreated, MBN+T7, and ALUI), and ADM9 cerebellum treatments (untreated, MBN+T7). Each peak is represented as a percentage of the first peak, the first peak being the start of the normal and expanded repeats combined. Additionally, the percent reduction in area under the peak from treated to untreated is given. Peak 9 is the location at which the normal allele ends and only the expanded allele is being scanned. (PDF) [file pgen.1003866.s006.pdf]
