# Supplementary material for: Detection of Slipped-DNAs at the Trinucleotide Repeats of the Myotonic Dystrophy Type I Disease Locus in Patient Tissues
Source: PLoS Genet. 2013 Dec 19;9(12):e1003866. doi: 10.1371/journal.pgen.1003866 (PMC3868534; doi:10.1371/journal.pgen.1003866)
Supplement: Figure S7 — Immunoprecipitated DNAs show structure by electron microscopy (EM). Electron microscopic analysis of slipped-DNAs. (A) As a positive control for slipped-DNA, in vitro-induced slipped-DNAs in the synthetic (CTG)800 repeat containing DNA fragments and its non-slipped variant were analyzed by EM. The fully-base paired (CTG)800 fragment appears with smooth contours (left panel), while the (CTG)800 fragments with induced slipped-DNAs appear thicker, with bends and kinked structures (right two panels). (B) The same multiple clustered short slip-outs were present in the immunoprecipitated DM1 patient DNAs (see also Fig. 5), consistent with previous EM results showing multiple bends in (CTG) repeat containing DNAs with induced slipped-DNAs [16]. (C) Quantification of slipped out molecules by tissue, as a percentage of total number of molecules seen in an EM field. Total number of molecules counted per tissue ranged from 6 to 32. A significantly increased number of molecules with slip-outs were identified in tissues showing high levels of CTG instability compared to the cerebellum, which showed the lowest level of instability (skeletal muscle vs. cerebellum, two-sided t-test; p = 0.005; pancreas vs. cerebellum, two-sided t-test; p = 0.0202; all unstable tissues (heart, liver, pancreas, cortex, skeletal muscle) vs. cerebellum; p = 0.0386). (PDF) [file pgen.1003866.s007.pdf]

(A)

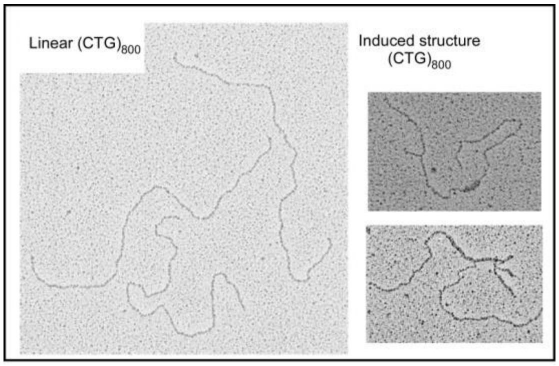

(B)

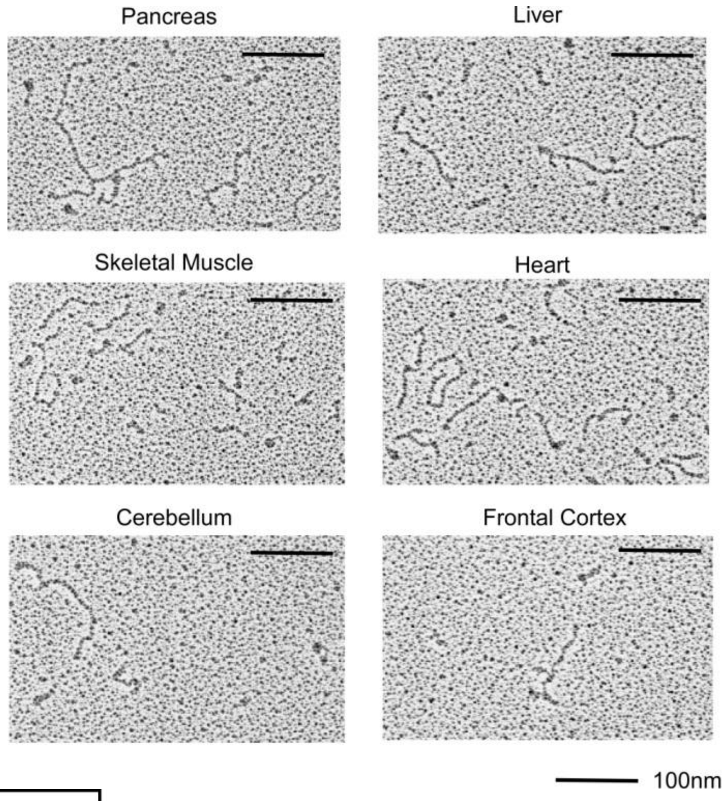

(C)

ADM5 Patient Tissue	Expanded Repeat #	% slipped molecules/ total molecules counted
Skeletal Muscle	3900-4400	87.5
Cortex	2900-5700	83
Pancreas	4900-5600	81
Liver	4600-5400	74
Heart	5100-6550	50
Cerebellum	1310	54

$p=0.005$

$p=0.0132$

$p=0.0202$

$p=0.038$
