# Supplementary material for: Detection of Slipped-DNAs at the Trinucleotide Repeats of the Myotonic Dystrophy Type I Disease Locus in Patient Tissues
Source: PLoS Genet. 2013 Dec 19;9(12):e1003866. doi: 10.1371/journal.pgen.1003866 (PMC3868534; doi:10.1371/journal.pgen.1003866)
Supplement: Table S1 — Primers used during study. This table details the sequences of the various primers used in this study. (PDF) [file pgen.1003866.s008.pdf]

Supporting Table 1. Primers used during study

Primer Name	Sequence 5' ->3'	Amplification Region
407	cagagcagggcgtcatgcaca	across DM1 repeat
409	gaagggtccttgtagccgggaa	
CTCFIIa	ctttctttcggccaggctgaggcc	across CTCFII site
CTCFIIb	aaagcaaatttcccgagtaagcaggc	
P3R	tacgcatcccagtttgagacg	along the DM1 triplet repeat (TP-PCR)
P4CTG	tacgcatcccagtttgagacgtgctgctgctgctgct	
Somy4R	cgggtttggcaaaagcaaatttcccga	
B13dx	gccagctgggtggtgataga	lamin-B2 non-DNA replication origin site
B13sx	cctcagaacccagctgtgga	

****** primer P3R binds to the tail of primer P4CTG. P4CTG is used in limiting quantities, such that only a small initial population of CTG-repeat PCR products are produced. P3R further amplifies those initial products.
