# Supplementary material for: Detection of Slipped-DNAs at the Trinucleotide Repeats of the Myotonic Dystrophy Type I Disease Locus in Patient Tissues
Source: PLoS Genet. 2013 Dec 19;9(12):e1003866. doi: 10.1371/journal.pgen.1003866 (PMC3868534; doi:10.1371/journal.pgen.1003866)
Supplement: Text S1 — Supporting notes and methods. This text describes in detail attributes of the reagents, materials, tissues, and assays used in this study, including, the Anti-DNA Junction Antibody (2D3), electron microscopic Imaging of immunoprecipitated DNAs, human tissues, DNA extraction procedures, CTG repeat length analysis, in vitro slipped-DNA structure formation, band-shift assay, DNA-immunoprecipitation, polymerase chain reaction protocols, mung bean nuclease and T7EndonucleaseI digestion, nuclear accessibility protocol, nucleosome assembly and disassembly on an expanded repeat-containing plasmid, and supporting references. (DOCX) [file pgen.1003866.s009.docx]

**Supporting Notes/Methods**

**Legend:**

**Supporting Notes/Methods**

**The Anti-DNA Junction Antibody (2D3)**

**Electron Microscopic Imaging of Immunoprecipitated DNAs**

**Human tissues**

**DNA extraction**

**CTG repeat length analysis**

**Slipped-DNA structure formation**

**Band-shift assay**

**DNA-Immunoprecipitation**

**Polymerase Chain Reaction Protocols**

**Mung Bean Nuclease and T7EndonucleaseI Digestion**

**Nuclear Accessibility Protocol (modified from [**[**42-44**](#_ENREF_42)**])**

**Electron Microscopy**

**Nucleosome Assembly and Disassembly on an Expanded Repeat Containing Plasmid**

**Supporting References**

**The Anti-DNA Junction Antibody (2D3)**

The monoclonal anti-DNA junction antibody, 2D3 (IgG_1_), binds specifically to junction-containing DNAs with no sequence preference [[8-11](#_ENREF_8)], binding strongest to slipped-CAG/CTG DNAs [[8](#_ENREF_8)]. Binding of 2D3 is independent of superhelical tension—a feature that allows us to omit crosslinking and permits purification of slipped-DNAs in linear molecules [[12](#_ENREF_12)]. The 2D3 binding site has been mapped to DNA junctions [[9](#_ENREF_9)] and 1-2 antibodies bind per DNA junction [[9-11](#_ENREF_9)]. 2D3 does not bind to junction-free linear duplexes, single-stranded DNA, Z-DNA (shown in (3) using supercoiled SV40 DNA, known to contain at least three Z-DNA forming tracts) or triple-stranded DNA; it is uniquely directed to 3-way and 4-way DNA junctions [[9-15](#_ENREF_9)]. There is no binding of 2D3 to a hairpin DNA in the presence of competitor DNA (3), and DNA footprinting showed that 2D3 binds at the base of the DNA junction, not to the hairpin tip (4). 2D3 has successfully purified cruciform-containing DNAs from human [[11](#_ENREF_11)] and yeast cells [[16](#_ENREF_16)]. Unlike those protocols, isolation of slipped-DNAs does not require binding antibody to supercoiled (unbroken) DNAs, as slipped-DNAs are very stable biophysically even in sheared DNAs, and survive DNA isolation, purification and de-proteinization [[2](#_ENREF_2)].

**Electron Microscopic Imaging of Immunoprecipitated DNAs**

The preparation of DNAs for electron microscopic analysis allows for high-resolution detection of DNA structures [[6](#_ENREF_6),[7](#_ENREF_7)]. We are able to readily assess slip-outs of 10-96 repeats. However, due to the resolution of the technique, smaller multiple slip-outs, often detected as kinks or bends, may be missed, because the manner in which they lie on the grid they do not generate kinks or bulges in the overall curvature of the molecules. Furthermore the thickening of the immunoprecipitated DNAs (2-3-fold thicker than typical fully-duplexed DNA) belies a high concentration of clustered short slip-outs [[6](#_ENREF_6),[7](#_ENREF_7)].

We are unclear as to why the size of the immunoprecipitated slipped-DNAs from the DM1 locus were smaller than expected for the range of sizes of the *Bbs*I-*Bam*HI restriction fragment containing CTG expansions (Fig. 2C). We suggest that during the DNA-IP process these very long CTG expansions with slip-outs may be sensitive to DNA shearing, a phenomenon previously observed for some but not all immunoprecipitations and specific DNA regions [[17-21](#_ENREF_17)]. Perhaps the increased shear sensitivity of the expanded repeats may be due either to the very long sizes of the expansions we studied, an enrichment of single-strand nicks or gaps or DNAse I hypersensitive sites in the expanded DM1 locus, which are known to vary over different genomic regions [[18-20](#_ENREF_18)] and be particularly sensitive to double-strand breakage, as previously noted for expanded CTG tracts [[22](#_ENREF_22)]. An enrichment of such strand breaks may be expected for arrested repair of clustered slip-out lesions in CTG/CAG tracts [[23](#_ENREF_23)] and CTG/CAG site fragility [[24](#_ENREF_24)].

**Human tissues**

Human tissues analysed in this study are listed in Fig. 2B. Autopsy tissues from a non-affected individual (ADN1) were obtained snap-frozen from the National Disease Research Interchange. Autopsy tissues from affected individuals were obtained as soon as possible post mortem, snap frozen, and stored at -80 degrees. Individual ADM5, a male with classical DM1, died at 55 years (died of aspiration pneumonia and respiratory failure), autopsy was performed 8 hours post-mortem. Individual ADM9, a female with juvenile DM1, died at 44 years (died of respiratory failure, had ovarian cancer), autopsy was performed 3 hours post-mortem. Non-DM1 individual ADN1, a male died of cardiac failure at 54 years, autopsy was performed 10 hours post-mortem.

**DNA extraction**

DNA extractions from human tissues were carried out to eliminate conditions that might be considered to allow unusual DNA structure formation and/or DNA shearing, using low binding tubes (Marsh Biomedical Products), as recommended[[25](#_ENREF_25),[26](#_ENREF_26)].Flash-frozen tissues were crushed by mortar and pestle in liquid nitrogen, and subsequently mixed with 10 x volume lysis buffer (50 mM Tris pH8, 100 mM EDTA, 100 mM NaCl, 0.1% SDS, 200 ug/ml proteinase K). This was incubated at 40°C overnight. The following day protein extraction was repeated two times with an equal volume of a 1:1 mixture of phenol and chloroform/isoamyl alcohol (24:1) (pH 8.0) and once with chloroform/isoamyl alcohol (pH 8.0) using a 10 ml serological pipette and pipetting slowly. Samples were mixed for 20 minutes at room temperature and centrifuged at 14000 rpm for 10 minutes after each phenol/chloroform/isoamyl alcohol extraction step, and mixed for 20 minutes and centrifuged at 14000 rpm for 5 minutes after the chloroform/isoamyl alcohol extraction step. DNA was precipitated by adding 1/10 volumes of 3 M NaOAc and an equal volume of isopropanol, then mixed gently until stringy DNA was visible. After centrifugation, the pellet was rinsed with 70% ethanol, then 95% ethanol. The pellet was air-dried for no more than 20 minutes (allows for the removal of excess ethanol but not drying of pellet), and wet pellets were resuspended in 1xTE (10 mM Tris, 0.1 mM EDTA, pH 8.0). Samples were allowed to hydrate for 10 minutes and placed at 40°C for two hours. To avoid denaturation, DNAs were never allowed to be dehydrated [[27](#_ENREF_27)]. In regular polypropylene tubes only low-levels of non-specific polypropylene-induced slipped-DNAs formed by CTG/CAG repeats was observed [[7](#_ENREF_7)],[[28](#_ENREF_28)]. DNA dehydration led to similar results[[27](#_ENREF_27)] and hence must be avoided. Our use of phenol to extract proteins was done under conditions known to deter against DNA denaturation and enhance the stability of the duplexed state. Specifically we used conditions of the Phenol Emulsion Reassociation Technique or PERT assay, which is widely used to enhance DNA renaturation [[29-32](#_ENREF_29)].

**CTG repeat length analysis**

Human-CTG repeat lengths were assessed by Southern blot using an LNA probe [DIG-labelled(CAG)7-5′-gcAgCagcAgCagCagcAgca-3′], as described previously [[5](#_ENREF_5)]. This sizing method takes advantage of the increased electrophoretic resolution of DM1 restriction fragments having a minimum of non-repetitive flanking sequences. Non-expanded CTG allele was sized by sequencing [The Centre for Applied Genomics (TCAG), MaRS Centre, Toronto, Canada] of the products obtained after PCR amplification (forward primer 409: 5′-gaagggtccttgtagccgggaa-3′; reverse primer 407: 5′-cagagcagggcgtcatgcaca-3′; 66.58°C annealing).

**Slipped-DNA structure formation**

Structures for band-shift assays were made as previously described [[6](#_ENREF_6)] with minor changes. Briefly, homoduplex slipped-structures (S-DNAs) of 30 or 50 repeat containing DNAs end-labeled with 32P were formed by alkaline denaturation/renaturation, described in detail in [[2](#_ENREF_2)]. Heteroduplex SI-DNAs were prepared as described[[6](#_ENREF_6)]. Briefly, DNAs of (CTG)50, (CAG)50, (CTG)47, (CAG)47, (CTG)49, (CAG)49 , (CTG)30, and (CAG)30 were end-labeled, mixed with an equimolar amount of a corresponding triplet repeat in order to get slip-outs of 20, 3, and 1 repeat, on both strands, and then heteroduplexed by denaturation/renaturation. Reduplexed plasmids were electrophoretically resolved on a 4% (w/v) polyacrylamide gel for 1.5 hours at a constant 200 V and then gel purified and then used in band-shift assays.

**Band-shift assay**

The radioactivity of each structure was determined using Cerenkov counting, and an equivalent radioactive concentration of each structure was run on a 4% (w/v) polyacrylamide gel in 1 x TBE buffer at a constant 150 V for 1.5 hours. Following electrophoresis gels were dried and autoradiographed. The negative control used for binding was an anti-actin antibody. Fully duplexed (FD) (CTG)50/(CAG)50 were used to test for specificity of binding, with linearized p-Bluescript plasmid additionally being used as a competitor during antibody binding.

**DNA-Immunoprecipitation**

Immunoprecipitation of slipped-structures in patient DNA was carried out using the anti-DNA junction monoclonal antibody 2D3. To minimize random shearing of the extensively expanded CTG-tract containing DNAs, we restriction digested 1 ug of genomic DNAs with *Bam*HI and *Bbs*I (see Fig. 2C) overnight at 37°C. 50 ul of hybridoma 2D3 culture supernatant containing ~5 ug/ml immunoglobulin was diluted 1:1 with PBS and added to the restriction digested patient DNA the following day and incubated on ice for one hour. 80 ul Protein G beads (Millipore, 0.2mg/ml) were prepared by washing once with 500 ul 0.15M NaCl and 10 mM Tris buffer (pH7.4) and once with 500 ul 1 x TE buffer (pH7.4), and resuspended in 250 ul 1 x TE. In order to extract antibody bound DNA, the resuspended beads were added to the antibody-DNA mixture and incubated on ice for 1 hour, mixing occasionally. The antibody/DNA/bead complex was washed free of unbound DNA three times with 1ml buffer containing 0.5M NaCl, 1 mg/ml bovine serum albumin, and 10 mM Tris (pH7.4) by centrifuging bead complex at 11000 rpm for 20 seconds, decanting supernatant, and gently resuspending the pellet in new wash solution. This was repeated with a buffer containing 0.15 M NaCl, 10 mM Tris (pH7.4) and 0.1% Nonidet-40. The affinity purified DNA was then eluted by resuspending the complex in 1 x TE buffer (pH 7.4) containing 2% SDS followed by centrifugation at 11000 rpm for 2 minutes. DNA was further purified by phenol-chloroform-isoamyl alcohol extraction, followed by 100% ethanol precipitation. Previous studies have found that DNA-immunoprecipitations, with few exceptions, can bring down DNA fragment sizes of 0.370-23 kb [[16](#_ENREF_16),[17](#_ENREF_17),[33](#_ENREF_33)], arguing against a possible bias against bringing down the larger CTG-expanded DM1 allele.

**Polymerase Chain Reaction Protocols**

Multiplex PCR analysis was carried out using primer sets 407-409 and CTCFIIa-CTCFIIb (sequences previously described in [[34](#_ENREF_34)] with an annealing temperature of 67°C. Products were resolved on a 4% non-denaturing acrylamide gel. Triplet-primed PCR analysis was carried out as described [[35-41](#_ENREF_35)], using primer set P3R, P4CTG, and Somy4R, with an annealing temperature of 62°C. Amplification of the *LaminB2* locus was carried out using primers B13dx and B13sx, with an annealing temperature of 64 degrees as previously described [[34](#_ENREF_34)].

**Mung Bean Nuclease and T7EndonucleaseI Digestion**

10 Units of MBN or T7endoI were incubated with 200 ng DM1 patient genomic, un-IP’d DNA in NEB Buffer 2 (New England Biolabs) at 30°C for 30 minutes and 37°C for 30 minutes, respectively. After digestion, the DNA was subjected to TP-PCR (see Table S1 for primers). Products of TP-PCR with a fluorescently labeled primer were analyzed by capillary electrophoresis at The Centre for Applied Genomics [TCAG- MaRS Centre, Toronto, Canada]. TP-PCR run with standard, non-fluorescent primers were run on a 1% agarose gel at a constant 100V and visualized under UV light. Restriction digestion of patient genomic DNA before IP was done with 100 units of *Bbs*I and *Bam*HI per ug of genomic DNA in NEB Buffer 2 (New England Biolabs) at 37°C overnight.

**Nuclear Accessibility Protocol (modified from [**[**42-44**](#_ENREF_42)**])**

Basic Buffer A was made as per [[42](#_ENREF_42)] but with the addition of 0.0 5M NaHSO3 to inhibit proteolysis and 0.5% Triton X to remove contaminating cytoplasmic membranes [[44](#_ENREF_44)]. Fresh frozen tissues were homogenized rapidly in 7ml/g of Buffer A (+ 0.34 M sucrose, 2 mM EDTA, 0.5 mM EGTA). Homogenates were layered on 0.33 volumes of Buffer A (+1.37 M sucrose, 1 mM EDTA, 0.25 mM EGTA) and centrifuged for 15 minutes at 16,000X g. The nuclear pellet was gently dispersed in 7 volumes Buffer A (+2.4 M sucrose, 0.1 mM EDTA, 0.1 mM EGTA), layered over an equal volume of the same buffer, and centrifuged for 45 minutes at 75,000Xg. The nuclear pellet was washed once in Buffer A (+ 0.34 M sucrose) by centrifuging for 15 minutes at 16,000Xg. The nuclei were then resuspended in 5% sucrose + 5mM Tris (pH7.4). Concentrations of nuclei were adjusted to 0.5 mg/ml (A260=10) using the appropriate enzyme reaction buffer (of either mung bean nuclease, T7 endonuclease, or *Alu*I (all from NEB). The mixtures were pre-incubated at the appropriate temperature for 5 minutes, and the enzyme was added (5000 units), and digested overnight. Genomic isolation was carried out as above, followed by TP-PCR.

**Electron Microscopy**

Immunoprecipitated DNA samples were analyzed by electron microscopy as described [[7](#_ENREF_7)]. Briefly, the indicated immunoprecipitated DNAs were mixed in a buffer containing 2 mM spermidine, adsorbed to glow-charged carbon-coated grids, washed with a water/graded ethanol series and rotary shadow cast with tungsten. Samples were examined using a Philips 400 electron microscope. The NIH Image J program was used to measure the length of DNA molecules.

**Nucleosome Assembly and Disassembly on an Expanded Repeat Containing Plasmid**

Nucleosome assembly was carried out as described (43) with modifications. Plasmid DNAs containing (CTG)250•(CAG)250 repeats were mixed with purified HeLa cell histone octamers [in ratios of 1:1, 1:2, or 1:4 (w/w)] in a buffer containing 2 M NaCl. The salt was slowly lowered in increments of 0.1 M to a final concentration of 0.1 M by adding a solution of 20 mM Hepes, 1 mM EDTA, pH 7.5 (5 min for each step at room temperature), to form stable nucleosomes. One portion of the nucleosome-assembled DNA was subjected to restriction enzyme digestion to verify nucleosome assembly. The remainder of the nucleosome assembled DNA was incubated with a solution containing protease K and SDS to remove histones, and then digested with *Hin*dIII and *Eco*RI to release the CTG-containing sequence. These digested plasmids was then run on a 1% agarose gel at 100V for 3 hours, along with a (CTG)250•(CAG)250 repeat-containing fragment from the same plasmid in which S-DNA had been induced for visual comparison.

**Supporting References**

1. Wang YH, Amirhaeri S, Kang S, Wells RD, Griffith JD (1994) Preferential nucleosome assembly at DNA triplet repeats from the myotonic dystrophy gene. Science 265: 669-671.

2. Pearson CE, Sinden RR (1996) Alternative structures in duplex DNA formed within the trinucleotide repeats of the myotonic dystrophy and fragile X loci. Biochemistry 35: 5041-5053.

3. Zentilin L, Giacca M (2010) The renaissance of competitive PCR as an accurate tool for precise nucleic acid quantification. Methods Mol Biol 630: 233-248.

4. Zentilin L, Giacca M (2007) Competitive PCR for precise nucleic acid quantification. Nat Protoc 2: 2092-2104.

5. Lopez Castel A, Nakamori M, Tome S, Chitayat D, Gourdon G, et al. (2011) Expanded CTG repeat demarcates a boundary for abnormal CpG methylation in myotonic dystrophy patient tissues. Hum Mol Genet 20: 1-15.

6. Pearson CE, Tam M, Wang YH, Montgomery SE, Dar AC, et al. (2002) Slipped-strand DNAs formed by long (CAG)*(CTG) repeats: slipped-out repeats and slip-out junctions. Nucleic Acids Res 30: 4534-4547.

7. Pearson CE, Wang YH, Griffith JD, Sinden RR (1998) Structural analysis of slipped-strand DNA (S-DNA) formed in (CTG)n. (CAG)n repeats from the myotonic dystrophy locus. Nucleic Acids Res 26: 816-823.

8. Tam M, Erin Montgomery S, Kekis M, Stollar BD, Price GB, et al. (2003) Slipped (CTG).(CAG) repeats of the myotonic dystrophy locus: surface probing with anti-DNA antibodies. J Mol Biol 332: 585-600.

9. Frappier L, Price GB, Martin RG, Zannis-Hadjopoulos M (1989) Characterization of the binding specificity of two anticruciform DNA monoclonal antibodies. J Biol Chem 264: 334-341.

10. Frappier L, Price GB, Martin RG, Zannis-Hadjopoulos M (1987) Monoclonal antibodies to cruciform DNA structures. J Mol Biol 193: 751-758.

11. Steinmetzer K, Zannis-Hadjopoulos M, Price GB (1995) Anti-cruciform monoclonal antibody and cruciform DNA interaction. J Mol Biol 254: 29-37.

12. Stollar BD (1986) Antibodies to DNA. CRC Crit Rev Biochem 20: 1-36.

13. Ward GK, Shihab-el-Deen A, Zannis-Hadjopoulos M, Price GB (1991) DNA cruciforms and the nuclear supporting structure. Exp Cell Res 195: 92-98.

14. Ward GK, McKenzie R, Zannis-Hadjopoulos M, Price GB (1990) The dynamic distribution and quantification of DNA cruciforms in eukaryotic nuclei. Exp Cell Res 188: 235-246.

15. Zannis-Hadjopoulos M, Frappier L, Khoury M, Price GB (1988) Effect of anti-cruciform DNA monoclonal antibodies on DNA replication. Embo J 7: 1837-1844.

16. Callejo M, Alvarez D, Price GB, Zannis-Hadjopoulos M (2002) The 14-3-3 protein homologues from Saccharomyces cerevisiae, Bmh1p and Bmh2p, have cruciform DNA-binding activity and associate in vivo with ARS307. J Biol Chem 277: 38416-38423.

17. Schwartz YB, Kahn TG, Pirrotta V (2005) Characteristic low density and shear sensitivity of cross-linked chromatin containing polycomb complexes. Mol Cell Biol 25: 432-439.

18. Spector DJ, Johnson JS, Baird NL, Engel DA (2003) Adenovirus type 5 DNA-protein complexes from formaldehyde cross-linked cells early after infection. Virology 312: 204-212.

19. Fernandez JL, Lopez-Fernandez C, Gosalvez J, Goyanes V (1996) Differential sensitivity of some human alphoid and classical satellite DNA regions from lymphocyte chromosomes to in situ exonuclease III digestion. Genome 39: 1210-1213.

20. Deininger PL (1983) Random subcloning of sonicated DNA: application to shotgun DNA sequence analysis. Anal Biochem 129: 216-223.

21. Reneker JS, Brotherton TW (1991) Discrete regions of the avian beta-globin gene cluster have tissue-specific hypersensitivity to cleavage by sonication in nuclei. Nucleic Acids Res 19: 4739-4745.

22. Hayward GS (1974) Unique double-stranded fragments of bacteriophage T5 DNA resulting from preferential shear-induced breakage at nicks. Proc Natl Acad Sci U S A 71: 2108-2112.

23. Panigrahi GB, Slean MM, Simard JP, Gileadi O, Pearson CE (2010) Isolated short CTG/CAG DNA slip-outs are repaired efficiently by hMutSbeta, but clustered slip-outs are poorly repaired. Proc Natl Acad Sci U S A 107: 12593-12598.

24. Freudenreich CH, Kantrow SM, Zakian VA (1998) Expansion and length-dependent fragility of CTG repeats in yeast. Science 279: 853-856.

25. Belotserkovskii BP, Johnston BH (1996) Polypropylene tube surfaces may induce denaturation and multimerization of DNA. Science 271: 222-223.

26. Belotserkovskii BP, Johnston BH (1997) Denaturation and association of DNA sequences by certain polypropylene surfaces. Anal Biochem 251: 251-262.

27. Svaren J, Inagami S, Lovegren E, Chalkley R (1987) DNA denatures upon drying after ethanol precipitation. Nucleic Acids Res 15: 8739-8754.

28. Pearson CE, Sinden, R.R. (1997) Slipped Strand DNA (S-DNA and SI-DNA) trinucleotide repeat instability and mismatch repair: A short review. In: Sarma RH, Sarma, R.H., editor. Biological Structure and Dynamics. New York: Adenine Press. pp. 191-207.

29. Pan-Hammarstrom Q, Wen S, Ghanaat-Pour H, Solders G, Forsberg H, et al. (2003) Lack of correlation between the reduction of serum immunoglobulin concentration and the CTG repeat expansion in patients with type 1 dystrophia [correction of Dystrofia] myotonica. J Neuroimmunol 144: 100-104.

30. Prior TW (2009) Technical standards and guidelines for myotonic dystrophy type 1 testing. Genet Med 11: 552-555.

31. Kuo HC, Hsieh YC, Wang HM, Chuang WL, Huang CC (2008) Correlation among subcortical white matter lesions, intelligence and CTG repeat expansion in classic myotonic dystrophy type 1. Acta Neurol Scand 117: 101-107.

32. Sabovic M, Medica I, Logar N, Mandic E, Zidar J, et al. (2003) Relation of CTG expansion and clinical variables to electrocardiogram conduction abnormalities and sudden death in patients with myotonic dystrophy. Neuromuscul Disord 13: 822-826.

33. Bell D, Sabloff M, Zannis-Hadjopoulos M, Price G (1991) Anti-cruciform DNA affinity purification of active mammalian origins of replication. Biochim Biophys Acta 1089: 299-308.

34. Cleary JD, Tome S, Lopez Castel A, Panigrahi GB, Foiry L, et al. (2010) Tissue- and age-specific DNA replication patterns at the CTG/CAG-expanded human myotonic dystrophy type 1 locus. Nat Struct Mol Biol 17: 1079-1087.

35. Hunter JM, Crouse AB, Lesort M, Johnson GV, Detloff PJ (2005) Verification of somatic CAG repeat expansion by pre-PCR fractionation. J Neurosci Methods 144: 11-17.

36. Falk M, Vojtiskova M, Lukas Z, Kroupova I, Froster U (2006) Simple procedure for automatic detection of unstable alleles in the myotonic dystrophy and Huntington's disease loci. Genet Test 10: 85-97.

37. Warner JP, Barron LH, Goudie D, Kelly K, Dow D, et al. (1996) A general method for the detection of large CAG repeat expansions by fluorescent PCR. J Med Genet 33: 1022-1026.

38. Kakourou G, Dhanjal S, Mamas T, Serhal P, Delhanty JD, et al. (2010) Modification of the triplet repeat primed polymerase chain reaction method for detection of the CTG repeat expansion in myotonic dystrophy type 1: application in preimplantation genetic diagnosis. Fertil Steril 94: 1674-1679.

39. Radvansky J, Ficek A, Minarik G, Palffy R, Kadasi L (2011) Effect of unexpected sequence interruptions to conventional PCR and repeat primed PCR in myotonic dystrophy type 1 testing. Diagn Mol Pathol 20: 48-51.

40. Addis M, Serrenti M, Meloni C, Cau M, Melis MA (2012) Triplet-primed PCR is more sensitive than southern blotting-long PCR for the diagnosis of myotonic dystrophy type1. Genet Test Mol Biomarkers 16: 1428-1431.

41. Santoro M, Masciullo M, Pietrobono R, Conte G, Modoni A, et al. (2013) Molecular, clinical, and muscle studies in myotonic dystrophy type 1 (DM1) associated with novel variant CCG expansions. J Neurol 260: 1245-1257.

42. Burgoyne LA, Wagar MA, Atkinson MR (1970) Calcium-dependent priming of DNA synthesis in isolated rat liver nuclei. Biochem Biophys Res Commun 39: 254-259.

43. Panyim S, Bilek D, Chalkley R (1971) An electrophoretic comparison of vertebrate histones. J Biol Chem 246: 4206-4215.

44. Gaubatz J, Ellis M, Chalkley R (1979) Nuclease digestion studies of mouse chromatin as a function of age. J Gerontol 34: 672-679.
